# A report on the mechanisms of sweeteners in four musculoskeletal disorders: Insights from network toxicology, molecular docking, and molecular dynamics simulations

**DOI:** 10.1371/journal.pone.0347282

**Published:** 2026-08-11

**Authors:** Tengyao Niu, Xiao Zhang, Ye Jia, Zhe Liu, Donghui Cao, Yanrong Tian, Haifeng Yuan

**Affiliations:** 1 Department of Spinal Orthopedics, General Hospital of Ningxia Medical University, Xingqing, Yinchuan, China; 2 Department of Basic Medicine, Liaoning University of Traditional Chinese Medicine, Huanggu District, Shenyang, Liaoning Province, China; Zydus Research Center, INDIA

## Abstract

Sweeteners are widely-studied food additives linked to metabolism and obesity, yet their musculoskeletal effects remain largely unknown.To investigate the potential effects of sweeteners on the musculoskeletal system, this study integrated network toxicology, molecular docking, and molecular dynamics simulations to systematically analyze the molecular associations between sweetener exposure and intervertebral disc degeneration, myositis, osteoarthritis, and osteoporosis. The results showed that sweeteners may affect musculoskeletal health by interfering with 35 core targets, which are primarily enriched in inflammatory responses, metabolic disorders, and related signaling pathways. Molecular docking revealed that sweeteners exhibited strong binding potential with these core targets, with mogroside and steviol glycosides showing particularly prominent predicted binding affinities. Molecular dynamics simulations further confirmed the binding stability of FASN, NOS2, and PCSK9 with these two sweeteners. This study provides a computational perspective on the potential disruption of musculoskeletal molecular networks by sweeteners, offering new targets and a theoretical basis for subsequent experimental validation and safety assessment.

## Introduction

Epidemiological and experimental studies have shown that added sugar consumption contributes to obesity and has detrimental effects on chronic diseases [1]. To address obesity and diet-related chronic conditions, multiple government and non-governmental health organizations have recommended limiting sugar intake [2,3]. As a result, many individuals—particularly those with chronic diseases—have turned to non-sugar sweeteners as alternatives [4]. Non-sugar sweeteners are low-calorie or calorie-free compounds, which can be either artificial or natural [5,6]. Artificial sweeteners are typically chemically synthesized and characterized by high sweetness intensity and minimal caloric content [6,7]. They undergo rigorous evaluation before being approved for use; six artificial sweeteners—aspartame, neotame, saccharin, acesulfame potassium (Ace-K), sucralose, and advantame—have been authorized by the U.S. Food and Drug Administration (FDA) as food additives [8]. Natural sweeteners, on the other hand, are extracted from plants and fruits, also offer very low caloric content, and are often perceived as healthier options [9]. Although sweeteners have contributed to body weight management and glycemic control, a growing body of evidence suggests that their consumption may lead to adverse metabolic effects [10]. For instance, they may disrupt gut microbiota metabolism, increasing the risk of inflammation; impair the beneficial functions of high-density lipoprotein (HDL), leading to loss of its antioxidant and anti-atherosclerotic activities; and elevate the risk of cancer [11,12]. As a key metabolic target organ system, the musculoskeletal system has naturally drawn attention. Some studies suggest that sweeteners exert neutral or even beneficial effects on muscle metabolism and bone structure, while others point to potential adverse outcomes, including alterations in metabolic pathways or impaired skeletal development [13]. Notably, some existing studies on sweeteners have indicated their adverse effects on musculoskeletal disorders, but their potential impact on bone health has not been clearly explored.

Musculoskeletal disorders (MSDs) encompass a group of conditions characterized by pain and functional limitations, and have become the leading cause of non-fatal disability worldwide [14]. According to the Global Burden of Disease Study 2021, approximately 169 million cases of MSDs were reported globally, contributing to 118,500 deaths and 161.9 million disability-adjusted life years (DALYs) [15]. To investigate the effects of sweeteners on these conditions, we focused on four representative disorders: intervertebral disc degeneration (IVDD), myositis, osteoarthritis (OA), and osteoporosis (OP) [16]. The development of these conditions is closely associated with genetic susceptibility, mechanical stress, aging, and inflammation [17].

IVDD is a naturally occurring process resulting from biological aging and lifelong exposure to normal physiological loads, characterized by loss of structural integrity in the intervertebral disc tissue [18]. Myositis refers to a group of autoimmune inflammatory muscle disorders characterized by progressive muscle weakness, immune cell infiltration, and muscle fiber necrosis, with major subtypes including polymyositis and dermatomyositis [19]. OA is a widespread chronic degenerative disease often accompanied by significant pain and disability, featuring degradation of cartilage and extracellular matrix (ECM), synovial inflammation, and subchondral bone remodeling [20]. OP is a systemic skeletal disease in which genetic and environmental factors collectively contribute to increased bone resorption, reduced bone formation, imbalanced bone remodeling, decreased bone mass, and heightened bone fragility [21]. This study employs network toxicology, molecular docking, and molecular dynamics simulations. It investigates the potential toxic mechanisms of sweeteners in musculoskeletal disorders. We aim to reveal the molecular basis of sweetener-induced musculoskeletal conditions. Specifically, we clarify the signaling pathways involved in disease onset and progression. These findings offer new insights into prevention and treatment strategies.

## Materials and methods

### Disease and sweeteners target library construction

Disease and sweetener targets were obtained from the GeneCards database (https://www.genecards.org/) and the Comparative Toxicogenomics Database (https://ctdbase.org/). The disease-related keywords used were “intervertebral disc degeneration”, “myositis”, “osteoarthritis”, and “osteoporosis”. The compound keyword used was “sweetener”. To ensure sufficient relevance to musculoskeletal diseases and sweetener effects, genes with a score above the median GeneCards score were selected. Targets from both databases were merged and duplicates were removed. This process generated a sweetener-specific target dataset and a disease-specific target dataset.

### Intersecting genes and PPI network construction

After identifying the intersecting genes, we uploaded them to the Search Tool for the Retrieval of Interacting Genes/Proteins (STRING) database (https://cn.string-db.org/) to construct a protein-protein interaction (PPI) network. The core target information was downloaded in TSV format. Then we imported the data into Cytoscape software(Cytoscape 3.10.4) to visualize the target gene network. Using the cytoHubba plugin, we identified the hub genes.

### GO and KEGG pathway analysis

Gene Ontology (GO) is an internationally standardized classification system for gene function. It describes the roles of genes and their products, including biological processes (BPs), molecular functions (MFs), and cellular components (CCs). The Kyoto Encyclopedia of Genes and Genomes (KEGG) is a database that integrates genomic, chemical, and systemic functional information. Its core component is KEGG PATHWAY, which maps molecular interactions and reaction networks. The overlapping genes from the Venn diagram analysis were uploaded to the Metascape database (https://metascape.org/) for GO and KEGG enrichment analyses. GO terms and KEGG pathways were ranked by p-value. The top 10 most significant GO terms and KEGG pathways were selected. The results were visualized using a bioinformatics online platform (https://bioinformatics.com.cn/).

### Molecular docking

We performed molecular docking between the hub genes common to the four diseases and various sweetener molecules. The hub genes were obtained from the Venn diagram analysis. For sweeteners, we selected five representative and commonly used natural sweeteners and five artificial sweeteners. These included allulose, erythritol, xylitol, mogroside, stevioside, acesulfame, aspartame, neotame, sucralose, and saccharin. They are internationally approved as food additives. All sweetener molecules have well-defined chemical structures suitable for molecular docking. Their three-dimensional structures are available in the Supplementary File. In this large-scale screening study, we employed a blind docking approach to conduct an unbiased exploration of binding sites on the protein surface. A total of 350 sweetener-protein pairs were analyzed. All protein PDB files are also provided in the Supplementary File. To ensure reliability, each compound-protein combination was docked nine times using independent random seeds. The conformation with the lowest binding energy from the repeated experiments was selected for subsequent analysis, as it represents the strongest potential interaction.

The key software tools used in this study included PubChem, UniProt, PyMOL, and AutoDock Vina (version AutoDockTools-1.5.7). The detailed workflow was as follows. First, ligand SDF structure files were downloaded from the PubChem database and converted to PDB format using OpenBabel GUI (version 3.1.1). Receptor proteins were then preprocessed in PyMOL(Version 3.1.6.1) by removing water molecules and unrelated ligands. The processed proteins and ligands were converted to PDBQT format in AutoDock Vina for blind docking analysis. After docking, the results were exported and visualized using PyMOL to generate three-dimensional structures of the protein-ligand complexes. This process enabled the identification of key residues involved in interactions and the visualization of binding forces, such as hydrogen bonds and hydrophobic interactions. It provided a structural perspective for elucidating the interaction mechanisms between compounds and proteins.

### Molecular dynamics simulation

To evaluate the binding stability and molecular flexibility of FASN, NOS2, and PCSK9 proteins with mogroside and stevioside, we performed molecular dynamics simulations using the Amber 24 software package. The simulation systems were constructed with the LEaP tool. Protein structures were loaded and described using the ff14SB force field. Compounds were parameterized with the GAFF force field. Each system was solvated in a TIP3P water box with a 10.0 Å buffer and neutralized by adding Na^+^ and Cl⁻ ions. A two-stage energy minimization was conducted to eliminate steric clashes. First, solvent and ions were optimized with restraints on protein backbones. Then, restraints were removed for full-system minimization using steepest descent and conjugate gradient methods. System equilibration followed two steps. The system was gradually heated from low temperature to 300.0 K under constant temperature with backbone restraints. Subsequently, equilibration continued under constant pressure to adjust density, maintaining 300.0 K and 1 bar.Finally, 100 ns production runs were performed under constant temperature (300.0 K) and pressure (1 bar) without restraints. Trajectories were analyzed using CPPTRAJ to calculate root mean square deviation (RMSD), radius of gyration (Rg), solvent accessible surface area (SASA), root mean square fluctuation (RMSF), and hydrogen bond numbers.

## Result

### The impact of sweetener on intervertebral disc degeneration

**Fig 1:**
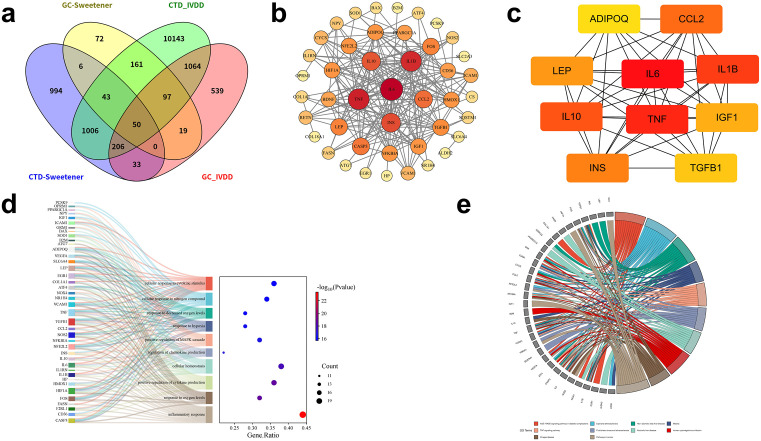
Integrative analysis of sweetener-related molecular networks and functional enrichment in intervertebral disc degeneration. (a) Venn diagram showing overlapping targets between IVDD and sweeteners. (b) Protein-protein interaction network of core overlapping targets. Hub genes are displayed as larger red nodes, with node size and color depth corresponding to their topological importance. (c) Top 10 hub genes identified by cytoHubba analysis. (d) GO enrichment analysis of core targets. (e) Top 10 KEGG pathways ranked by p-value, illustrating pathway-hub gene interactions.

To elucidate the molecular mechanisms linking sweetener exposure to intervertebral disc degeneration, we integrated bioinformatics analyses to identify overlapping targets and pathways. A total of 50 potential targets were identified (Fig. 1a). A protein-protein interaction network was constructed, revealing key nodes. IL-6, TNF, IL-1B, IL-10, and CCL2 were primarily involved in inflammatory responses. INS was mainly associated with blood glucose regulation (Fig. 1b). The top ten hub genes were screened using the CytoHubba plugin and classified by function. Inflammatory response genes included TNF, IL-6, IL-1B, IL-10, TGFB1, and CCL2. Energy metabolism and growth-related genes included LEP, INS, IGF1, and ADIPOQ (Fig. 1c).Functional annotation of the 50 core targets was performed using the DAVID database with *Homo sapiens* as the selected species. A total of 1,460 Gene Ontology (GO) terms were identified, including 1,313 biological processes (BPs), 82 cellular components (CCs), and 65 molecular functions (MFs). Additionally, 99 Kyoto Encyclopedia of Genes and Genomes (KEGG) pathways were enriched. For GO enrichment, the top ten terms with the highest gene ratios in BP, CC, and MF categories were visualized using bubble charts (Fig. 1d). For KEGG enrichment, the top ten pathways were presented in a chord diagram (Fig. 1e). GO enrichment showed that intersecting genes were mainly involved in biological processes such as cellular response to cytokine stimulus, neurological system processes, and cellular response to lipids. These processes are closely related to inflammation, neural regulation, and metabolism. KEGG enrichment indicated that pathways were primarily enriched in the AGE-RAGE signaling pathway, lipid and atherosclerosis, and non-alcoholic fatty liver disease. This suggests a major impact on inflammatory and lipid metabolism pathways. These findings clarify the pathways through which sweeteners may contribute to the development of intervertebral disc degeneration.

### The impact of sweetener on myositis

**Fig 2:**
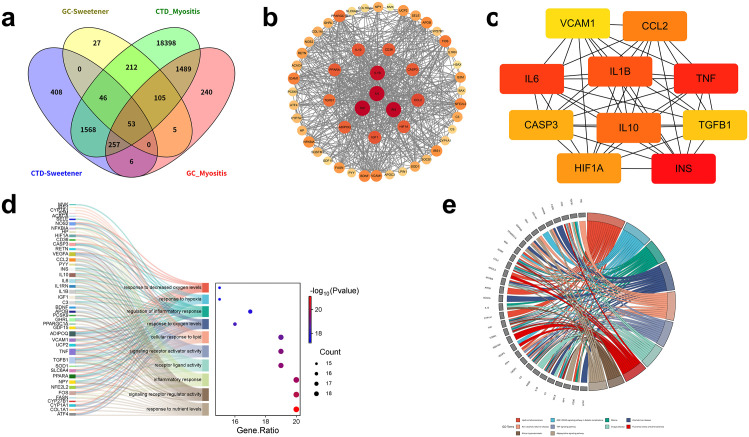
Integrative analysis of sweetener-related molecular networks and functional enrichment in myositis. (a) Venn diagram showing overlapping targets between myositis and sweeteners. (b) Protein-protein interaction network of core overlapping targets. Hub genes are displayed as larger red nodes, with node size and color depth corresponding to their topological importance. (c) Top 10 hub genes identified by cytoHubba analysis. (d) GO enrichment analysis of core targets. (e) Top 10 KEGG pathways ranked by p-value, illustrating pathway-hub gene interactions.

To elucidate the molecular mechanisms linking sweetener exposure to sarcopenia, we integrated bioinformatics analyses to identify overlapping targets and pathways. A total of 53 potential targets were identified (Fig. 2a). A protein-protein interaction network was constructed, revealing key nodes. These included inflammatory response genes (TNF, IL-6, IL-1B, IL-10, CCL2), energy metabolism and growth-related genes (INS, ADIPOQ, PPARA, IGF-1, CD36), cellular stress response genes (CASP3, HIF1A, TGFB1), and a lipid metabolism gene (CD36) (Fig. 2b). The top ten hub genes were screened using the CytoHubba plugin and classified by function. Inflammatory response genes included IL1B, TNF, IL6, IL10, CCL2, and VCAM1. Cellular stress genes included CASP3, HIF1A, and TGFB1. The energy metabolism gene was INS (Fig. 2c).Functional annotation of the 53 core targets was performed using the DAVID database with *Homo sapiens* as the selected species. A total of 1,505 Gene Ontology (GO) terms were identified, including 1,353 biological processes (BPs), 82 cellular components (CCs), and 70 molecular functions (MFs). Additionally, 105 Kyoto Encyclopedia of Genes and Genomes (KEGG) pathways were enriched. GO enrichment analysis showed that genes common to sweeteners and sarcopenia were mainly enriched in three major biological process categories: hypoxic stress and adaptation (response to decreased oxygen levels, response to hypoxia), inflammation and immune regulation (inflammatory response, regulation of inflammatory response), and metabolism and nutrition (response to nutrient levels, cellular response to lipid) (Fig. 2d). KEGG analysis indicated that intersecting genes were primarily enriched in pathways related to lipid and atherosclerosis, non-alcoholic fatty liver disease, the AGE-RAGE signaling pathway, and the TNF signaling pathway (Fig. 2e).

### The impact of sweetener on osteoarthritis

**Fig 3:**
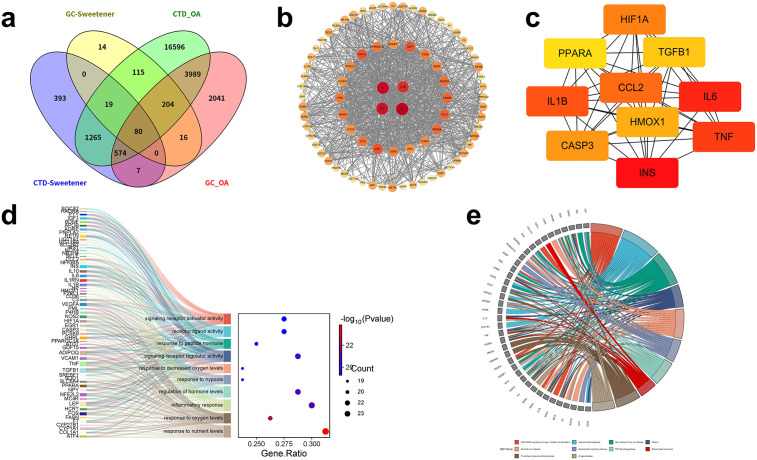
Integrative analysis of sweetener-related molecular networks and functional enrichment in osteoarthritis. (a) Venn diagram showing overlapping targets between OA and sweeteners. (b) Protein-protein interaction network of core overlapping targets. Hub genes are displayed as larger red nodes, with node size and color depth corresponding to their topological importance. (c) Top 10 hub genes identified by cytoHubba analysis. (d) GO enrichment analysis of core targets. (e) Top 10 KEGG pathways ranked by p-value, illustrating pathway-hub gene interactions.

To investigate the molecular mechanisms linking sweetener exposure to osteoarthritis, we integrated bioinformatics analyses to identify overlapping targets and pathways. A total of 80 potential targets were identified (Fig. 3a). A protein-protein interaction network was constructed, revealing key nodes. These included inflammatory response genes (IL-6, IL1B, TNF, CCL2, IL-10), energy metabolism regulation genes (INS, IGF1, ADIPOQ, LEP, PPARA), and cellular stress response genes (HIF1A, CASP3, FOS, HMOX1) (Fig. 3b). The top ten hub genes were screened using the CytoHubba plugin and classified by function. These included inflammation and immune regulation genes (IL-6, TNF, IL-1B, CCL2), energy metabolism regulation genes (INS, PPARA), and cellular stress response genes (HIF1A, HMOX1, TGFB1, CASP3) (Fig. 3c). Functional annotation of the 80 core targets was performed using the DAVID database with *Homo sapiens* as the selected species. A total of 1,624 Gene Ontology (GO) terms were identified, including 1,429 biological processes (BPs), 104 cellular components (CCs), and 91 molecular functions (MFs). Additionally, 104 Kyoto Encyclopedia of Genes and Genomes (KEGG) pathways were enriched. GO enrichment analysis showed that intersecting genes were mainly enriched in endocrine and metabolism processes (response to peptide hormone, regulation of hormone levels), hypoxic stress response (response to decreased oxygen levels, response to hypoxia), and inflammatory response (Fig. 3d). KEGG analysis indicated that common genes were primarily enriched in lipid metabolism pathways (Lipid and atherosclerosis, Non-alcoholic fatty liver disease, Alcoholic liver disease, Adipocytokine signaling pathway) and inflammatory immune response pathways (AGE-RAGE signaling pathway in diabetic complications, TNF signaling pathway). These findings suggest that the mechanism of sweeteners in osteoarthritis operates through a multi-pathway synergistic network rooted in “metabolic disturbance → chronic inflammation → tissue stress and injury” (Fig. 3e).

### The impact of sweetener on osteoporosis

**Fig 4:**
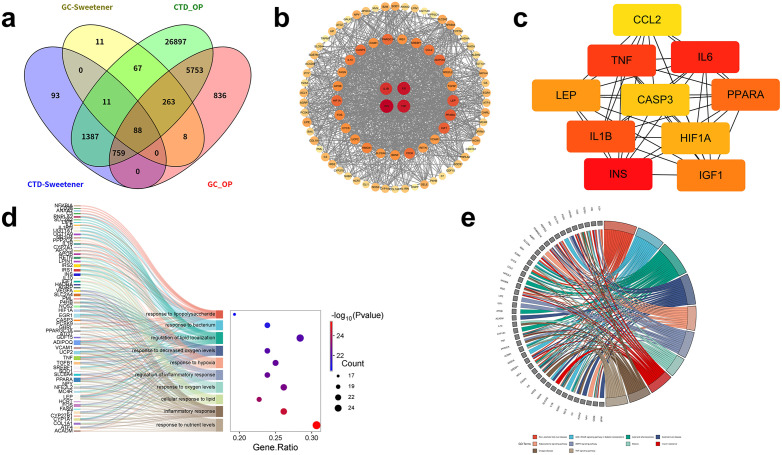
Integrative analysis of sweetener-related molecular networks and functional enrichment in osteoporosis. (a) Venn diagram showing overlapping targets between OP and sweeteners. (b) Protein-protein interaction network of core overlapping targets. Hub genes are displayed as larger red nodes, with node size and color depth corresponding to their topological importance. (c) Top 10 hub genes identified by cytoHubba analysis. (d) GO enrichment analysis of core targets. (e) Top 10 KEGG pathways ranked by p-value, illustrating pathway-hub gene interactions.

To investigate the molecular mechanisms linking sweetener exposure to osteoporosis, we integrated bioinformatics analyses to identify overlapping targets and pathways. A total of 88 potential targets were identified (Fig. 4a). A protein-protein interaction network was constructed, revealing key nodes. These included energy metabolism genes (INS, IGF1, LEP, ADIPOQ, PPARA, PPARGC1A, CD36, FASN, SREBF1), inflammatory response genes (IL6, IL1B, TNF, IL-10, CCL2), and cellular stress response genes (HIF1A, CASP3, HMOX1, FOS) (Fig. 4b).The top ten hub genes were screened using the CytoHubba plugin and classified by function. These included inflammatory response genes (TNF, IL-6, IL1B, CCL2), energy metabolism regulation genes (LEP, INS, IGF1, PPARA), and cellular stress response genes (HIF1A, CASP3) (Fig. 4c). Functional annotation of the 88 core targets was performed using the DAVID database with *Homo sapiens* as the selected species. A total of 1,744 Gene Ontology (GO) terms were identified, including 1,443 biological processes (BPs), 105 cellular components (CCs), and 89 molecular functions (MFs). Additionally, 107 Kyoto Encyclopedia of Genes and Genomes (KEGG) pathways were enriched. As shown in Fig. 4d, GO enrichment analysis was primarily concentrated in inflammatory response (response to lipopolysaccharide, inflammatory response); cellular stress, apoptosis, and cell death (apoptotic process, response to oxidative stress); and energy metabolism dysregulation (response to nutrient levels, regulation of developmental growth). KEGG enrichment analysis indicated that intersecting genes were mainly enriched in energy metabolism regulation pathways (Insulin resistance, Adipocytokine signaling pathway, AMPK signaling pathway, Non-alcoholic fatty liver disease); inflammatory response pathways (TNF signaling pathway, Malaria, Chagas disease); and end-target organ damage pathways (AGE-RAGE signaling pathway in diabetic complications, Lipid and atherosclerosis) (Fig. 4e).

### Molecular docking of proteins related to musculoskeletal system diseases with sweeteners

**Fig 5:**
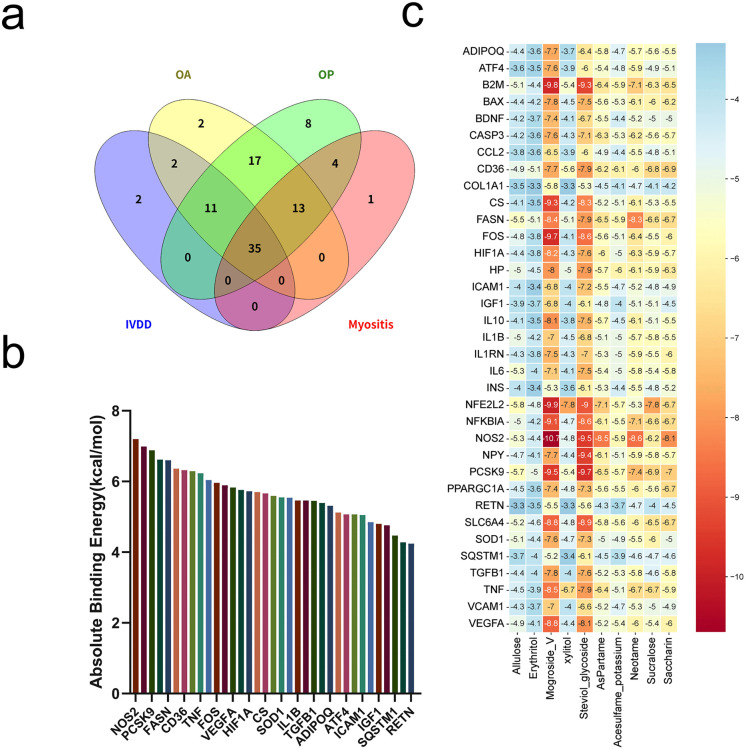
Molecular docking analysis of sweeteners with core musculoskeletal disease-related targets. (a) Four-way Venn diagram showing 35 overlapping target genes associated with sweetener exposure and four musculoskeletal pathologies (IVDD, myositis, osteoarthritis, and osteoporosis). (b) Heatmap of binding affinities between 10 sweeteners (rows) and 35 core targets (columns). Colors are scaled by docking energy (kcal/mol). (c) Ranked average binding energies of 35 targets with 10 sweeteners. Targets are ordered by average binding energy.

**Fig 6:**
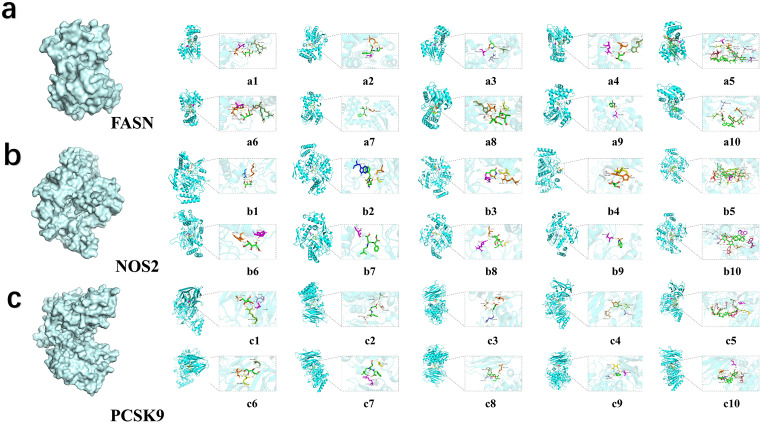
Visualization of molecular docking results between sweeteners and core targets FASN, NOS2, and PCSK9. (a) Docking results of FASN with 10 sweetener molecules: (a1) allulose, (a2) aspartame, (a3) acesulfame, (a4) erythritol, (a5) mogroside, (a6) xylitol, (a7) neotame, (a8) sucralose, (a9) saccharin, (a10) stevioside. (b) Docking results of NOS2 with 10 sweetener molecules: (b1) allulose, (b2) aspartame, (b3) acesulfame, (b4) erythritol, (b5) mogroside, (b6) xylitol, (b7) neotame, (b8) sucralose, (b9) saccharin, (b10) stevioside. (c) Docking results of PCSK9 with 10 sweetener molecules: (c1) allulose, (c2) aspartame, (c3) acesulfame, (c4) erythritol, (c5) mogroside, (c6) xylitol, (c7) neotame, (c8) sucralose, (c9) saccharin, (c10) stevioside.

To elucidate the shared molecular mechanisms of sweeteners across four musculoskeletal disorders, we performed a four-way Venn diagram analysis of their intersecting targets (Fig. 5a). This analysis revealed 35 core overlapping genes, all of which are protein-coding genes. To validate the interactions between these 35 gene targets and the 10 sweeteners, we employed molecular docking. This technique predicts binding affinity and interaction patterns at the molecular level, thereby revealing potential toxicological mechanisms. We assessed the interactions between the 10 sweeteners and the protein products of the 35 genes. The binding performance for each protein-sweetener pair was expressed as the average binding energy. A total of 350 molecular docking runs were performed, generating a dataset of 350 binding energy values (Fig. 5b). Based on average binding energy, we ranked the affinity of the 10 common natural and artificial sweeteners against the core targets (Fig. 5c). The results showed that mogroside and stevioside exhibited the lowest binding energies, indicating the strongest binding affinity. They were followed by aspartame, neotame, saccharin, sucralose, and acesulfame. In contrast, allulose, xylitol, and erythritol showed relatively higher binding energies and weaker affinity. To illustrate the molecular basis of these high-affinity interactions, we performed visual analysis of the binding modes for the top three core target complexes based on binding energy ranking (Fig. 6). The results showed that the ligand molecules were fully embedded within the binding pockets of the targets. Their excellent binding ability stemmed from a dense hydrogen bond network formed with key residues (e.g., GLU-332, GLN-302), tight encapsulation by a hydrophobic cavity (e.g., VAL-333), and favorable shape complementarity. These microscopic interactions collectively explain the docking screening rankings from a structural perspective. Overall, these findings not only identify key molecular targets for sweetener toxicity research but also reveal their binding structures, highlighting critical molecular targets through which sweeteners may affect the musculoskeletal system.

### Validation of Sweetener-Protein Interactions and Analysis of Binding Sites in Conserved Sequences

**Fig 7:**
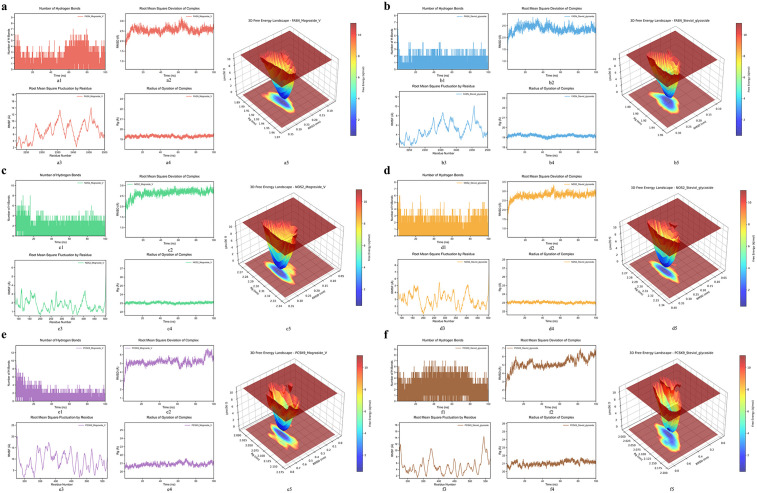
Molecular dynamics analysis of FASN, NOS2, and PCSK9 with mogroside and stevioside. (a) Molecular dynamics analysis of FASN with mogroside: (a1) hydrogen bond analysis between FASN and mogroside; (a2) root mean square deviation (RMSD) analysis between FASN and mogroside; (a3) root mean square fluctuation (RMSF) analysis between FASN and mogroside; (a4) radius of gyration (Rg) analysis between FASN and mogroside; (a5) free energy landscape analysis of the FASN-mogroside complex. (b) Molecular dynamics analysis of FASN with stevioside: (b1) hydrogen bond analysis between FASN and stevioside; (b2) RMSD analysis between FASN and stevioside; (b3) RMSF analysis between FASN and stevioside; (b4) Rg analysis between FASN and stevioside; (b5) free energy landscape analysis of the FASN-stevioside complex. (c) Molecular dynamics analysis of NOS2 with mogroside: (c1) hydrogen bond analysis between NOS2 and mogroside; (c2) RMSD analysis between NOS2 and mogroside; (c3) RMSF analysis between NOS2 and mogroside; (c4) Rg analysis between NOS2 and mogroside; (c5) free energy landscape analysis of the NOS2-mogroside complex. (d) Molecular dynamics analysis of NOS2 with stevioside: (d1) hydrogen bond analysis between NOS2 and stevioside; (d2) RMSD analysis between NOS2 and stevioside; (d3) RMSF analysis between NOS2 and stevioside; (d4) Rg analysis between NOS2 and stevioside; (d5) free energy landscape analysis of the NOS2-stevioside complex. (e) Molecular dynamics analysis of PCSK9 with mogroside: (e1) hydrogen bond analysis between PCSK9 and mogroside; (e2) RMSD analysis between PCSK9 and mogroside; (e3) RMSF analysis between PCSK9 and mogroside; (e4) Rg analysis between PCSK9 and mogroside; (e5) free energy landscape analysis of the PCSK9-mogroside complex. (f) Molecular dynamics analysis of PCSK9 with stevioside: (f1) hydrogen bond analysis between PCSK9 and stevioside; (f2) RMSD analysis between PCSK9 and stevioside; (f3) RMSF analysis between PCSK9 and stevioside; (f4) Rg analysis between PCSK9 and stevioside; (f5) free energy landscape analysis of the PCSK9-stevioside complex.

We ranked the average binding energies between the core targets and the 10 sweeteners. The top three core targets selected were NOS2, PCSK9, and FASN. From the sweetener perspective, stevioside and mogroside exhibited higher binding energies with these core targets. Therefore, stevioside and mogroside were selected for molecular dynamics simulations with the core targets. To investigate hydrogen bonding properties at the complex binding sites, we calculated the number of stable hydrogen bonds between ligands and proteins. Results showed that hydrogen bond numbers between core targets and compounds fluctuated minimally throughout the simulation, consistently ranging from 2 to 8. This indicates stable hydrophilic interactions (Fig. 7). Root mean square deviation (RMSD) is a key parameter for assessing protein-ligand complex stability. A flatter RMSD curve indicates greater complex stability. Results showed that RMSD curves for protein-compound complexes fluctuated within 2 Å with no significant deviations, demonstrating high stability (Fig. 7b). Root mean square fluctuation (RMSF) reflects the fluctuation degree of amino acid residues during simulation. Higher RMSF values indicate greater residue mobility, while lower values suggest restricted movement. Solvent accessible surface area (SASA) measures the contact area between protein surface and solvent. It is an important parameter for studying protein stability, interactions, and folding. Hydrophobic interactions are the primary driving force for protein folding, while solvent-contact regions are typically polar with weaker hydrophobic effects. Results showed notable SASA increases around 20 ns and 80 ns, indicating enhanced surface-solvent interactions that may affect solubility, reactivity, and bioactivity (Fig. 7c). Radius of gyration (Rg) describes protein structural compactness. Smaller Rg values indicate more compact and stable protein structures. Rg curves from molecular dynamics simulations showed stable performance throughout with minimal fluctuation, consistent with RMSD stability. Slight fluctuations observed near the simulation endpoint (around 80 ns) aligned with SASA analysis, indicating overall good complex stability (Fig. 7d). Free energy landscape projection in two-dimensional space (RMSD vs. Rg) during molecular dynamics simulations revealed conformational stability and dynamic behavior of protein-compound complexes. In the plots, RMSD represents deviation from initial structure, Rg reflects molecular compactness, and free energy (G, kJ/mol) quantifies thermodynamic stability of conformational states. Low-energy regions (dark blue) correspond to high-probability stable conformations, typically representing energy minima. High-energy regions (red) correspond to unstable transition states or low-probability conformations. Contour lines projected on the bottom highlight dense free energy distribution areas, helping identify major conformational clusters and their distribution. By analyzing free energy landscapes, we identified stable conformations within specific RMSD and Rg ranges, providing thermodynamic evidence for understanding binding mechanisms and structural dynamics (Fig. 7e). Through molecular dynamics simulations and subsequent analyses, protein-compound complexes demonstrated high stability. RMSD and Rg analyses showed minimal structural fluctuations with compact complex structures. RMSF indicated stable amino acid residue movement. SASA analysis revealed changes in solvent interactions. Hydrogen bond analysis confirmed stable hydrophilic binding. MM/GBSA analysis further quantified binding free energies, indicating that van der Waals forces and electrostatic interactions were the primary driving forces for complex stability. These findings provide insights into protein-compound binding mechanisms and offer theoretical basis for future drug design and optimization.

## Discussion

Overconsumption of sweeteners can lead to adverse metabolic effects. It may cause gut microbiota dysbiosis, increase the likelihood of inflammatory responses, and impair the beneficial functions of high-density lipoprotein (HDL) [22,23]. This results in loss of antioxidant and anti-atherosclerotic activity, potentially increasing cancer risk. Some studies also suggest that sweeteners may affect metabolic pathways, leading to impaired skeletal development.

**Fig 8:**
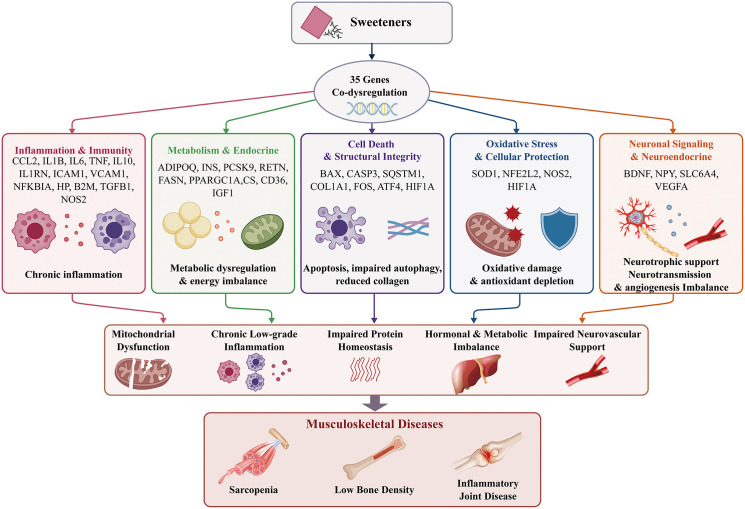
Functional classification of sweetener-related genes in musculoskeletal disorders.

Our study did not focus on a single compound or a single target. Instead, we systematically investigated the molecular mechanisms and regulatory networks through which multiple sweeteners act on genes common to musculoskeletal disorders. This approach provides a systemic, multi-target perspective on how sweeteners affect the musculoskeletal system. In the initial phase, we integrated and identified a set of key molecular regulators potentially disrupted by sweetener exposure. These regulators may play important roles in the pathogenesis of musculoskeletal disorders. These key targets represent not only core components in the toxicological progression from exposure to disease but also potential candidates for future therapeutic exploration. These targets are distributed across different pathways. Therefore, we performed systematic classification and functional annotation to elucidate their potential roles in sweetener-induced musculoskeletal disorders. Adiponectin(ADIPOQ) secretes adipokines and improves insulin sensitivity. Reduced levels are associated with obesity, insulin resistance, and abnormal bone metabolism [24]. Activating Transcription Factor 4 (ATF4) regulates endoplasmic reticulum stress and amino acid metabolism. It plays a key role in osteoblast differentiation and skeletal development [25]. Beta-2-microglobulin (B2M) is associated with inflammatory joint disease and dialysis-related amyloidosis. BCL2 Associated X Protein (BAX) induces changes in mitochondrial membrane permeability. It plays a role in chondrocyte and osteoblast apoptosis [26]. Brain-Derived Neurotrophic Factor (BDNF) promotes neuronal survival, regulates neuroplasticity and pain perception, and participates in bone metabolism and muscle regeneration [27]. Caspase-3 (CASP3) is an executioner protein in apoptosis. It is a key downstream effector in cell death pathways [28]. Chemokine ligand 2 (CCL2) drives chronic inflammation. It is closely associated with arthritis and intervertebral disc degeneration [29]. CD36 Molecule (CD36) participates in lipid metabolism and inflammatory signaling. It links metabolic disorders to tissue inflammation [30]. Collagen Type I Alpha 1 Chain (COL1A1) is a major structural protein in connective tissues such as bone and tendon. Citrate Synthase (CS) is the rate-limiting enzyme in the tricarboxylic acid cycle. It reflects mitochondrial function and cellular energy metabolism. Fatty Acid Synthase (FASN) catalyzes de novo fatty acid synthesis. It plays a role in energy storage and inflammatory signaling [31]. Fos Proto-Oncogene (FOS) responds to growth factors and stress signals. It regulates cell proliferation and apoptosis [32]. Hypoxia-Inducible Factor 1 Alpha (HIF1A) is a hypoxia-inducible factor. It regulates cellular adaptation to low oxygen, affecting angiogenesis, metabolism, and inflammation. Haptoglobin (HP) is an acute-phase response protein. It participates in antioxidant and inflammatory processes. Intercellular Adhesion Molecule 1 (ICAM1) mediates leukocyte adhesion and transendothelial migration. It amplifies local inflammatory responses. Insulin-like Growth Factor 1 (IGF1) promotes cell growth and proliferation. It stimulates skeletal growth, cartilage repair, and muscle protein synthesis [33]. Interleukin-10 (IL10) is a potent anti-inflammatory cytokine. It inhibits pro-inflammatory factor production and regulates immune homeostasis. Interleukin-1 Beta (IL1B) is a key pro-inflammatory cytokine. It drives inflammation, fever, and tissue destruction, serving as a core mediator in many inflammatory diseases. Interleukin-1 Receptor Antagonist (IL1RN) is a natural anti-inflammatory molecule. It functions through competitive inhibition of IL1B signaling [34]. Interleukin-6 (IL6) regulates immunity, acute-phase responses, metabolism, and hematopoiesis. It is persistently elevated in chronic inflammation. Insulin (INS) is a core hormone regulating glucose, lipid, and protein metabolism. Nuclear Factor Erythroid 2 Like 2 (NFE2L2) is a master transcription factor regulating antioxidant response elements. It activates antioxidant and detoxification gene expression [35]. NFKB Inhibitor Alpha (NFKBIA) inhibits NF-κB nuclear translocation. Its degradation is a key step in activating the NF-κB inflammatory pathway [36]. Nitric Oxide Synthase 2 (NOS2) is an inducible nitric oxide synthase. It produces large amounts of NO, participating in inflammation and oxidative damage. Neuropeptide Y (NPY) regulates appetite, energy balance, stress responses, and vasoconstriction. Proprotein Convertase Subtilisin/Kexin Type 9 (PCSK9) promotes LDL receptor degradation in hepatocytes, elevating plasma LDL-C levels. It is associated with cardiovascular risk [37]. Peroxisome Proliferator-Activated Receptor Gamma Coactivator 1 Alpha (PPARGC1A) is a major regulator of mitochondrial biogenesis and energy metabolism. It affects muscle function and insulin sensitivity. Resistin (RETN) is closely associated with insulin resistance and chronic inflammation. Solute Carrier Family 6 Member 4 (SLC6A4) regulates synaptic serotonin concentration. It influences mood, pain perception, and bone metabolism. Superoxide Dismutase 1 (SOD1) scavenges superoxide radicals. It is a key intracellular antioxidant enzyme [38]. Sequestosome 1 (SQSTM1) is a selective autophagy adaptor protein. It also functions as a scaffold protein in NF-κB signaling. Transforming Growth Factor Beta 1 (TGFB1) regulates cell growth, differentiation, immunity, and extracellular matrix synthesis. It plays dual roles in bone repair and fibrosis. Tumor Necrosis Factor (TNF) is a core pro-inflammatory cytokine. It regulates inflammation, apoptosis, and metabolism, serving as a therapeutic target in many autoimmune diseases. Vascular Cell Adhesion Molecule 1 (VCAM1) is a vascular cell adhesion molecule. It mediates adhesion of lymphocytes and monocytes to endothelial cells [39]. Vascular Endothelial Growth Factor A (VEGFA) stimulates angiogenesis. It is essential for skeletal growth, endochondral ossification, and tissue repair. In summary, we integrated key regulatory factors into a functional map, comprehensively revealing the molecular network potentially induced by sweetener exposure (Fig. 8). To enable an unbiased exploration of the protein surface, systematically scan the entire protein, and discover both allosteric sites distant from the active center and entirely new binding pockets, we employed blind docking between 10 sweeteners and 35 core targets, and subsequently calculated their binding energies. Results showed that the 35 core targets exhibited high binding affinities with multiple sweeteners. In the context of molecular docking, a more negative binding energy (e.g., ΔG ≤ −7.0 kcal/mol) indicates a stronger binding affinity between the ligand and the receptor [40]. Mogroside and stevioside displayed the lowest binding energies, indicating the strongest binding affinity. They were followed by aspartame, neotame, saccharin, sucralose, and acesulfame. In contrast, allulose, xylitol, and erythritol showed relatively higher binding energies with weaker affinity. These findings demonstrate that sweeteners may induce musculoskeletal disorders through these core targets. Enrichment analysis revealed that sweeteners affect the four diseases primarily through inflammatory responses, energy and metabolic pathways, cellular stress, and lipid metabolism. This indicates that the impact of sweeteners on musculoskeletal diseases operates through a synergistic multi-pathway network rooted in “metabolic disturbance → chronic inflammation → tissue stress and injury. “Based on strong binding affinities, we performed detailed analyses of FASN, NOS2, and PCSK9. Molecular docking results were visualized, demonstrating that ligand molecules were fully embedded within target binding pockets. This superior binding ability stemmed from a dense hydrogen bond network formed with key residues (e.g., GLU-332, GLN-302), tight encapsulation by a hydrophobic cavity (e.g., VAL-333), and favorable shape complementarity. We then selected mogroside and stevioside for molecular dynamics simulations with these targets. The compounds demonstrated stable binding with core targets throughout the simulations. This confirms the reliability of the molecular docking results and further validates the strong binding capacity between compounds and core targets. Importantly, this study does not dismiss the potential relevance of genes with weaker binding interactions or those less studied in this context. Our aim is to propose a foundational framework. In this study, blind docking revealed theoretical binding potential. However, according to the principle of “the dose makes the poison”, this potential does not necessarily translate into biological risk under realistic intake levels [41]. The strength of our approach lies in systematically scanning the entire protein surface and successfully identifying allosteric sites distant from the active center as well as novel binding pockets. Nevertheless, the calculated binding energies represent theoretical values under ideal conditions rather than direct evidence of *in vivo* effects. The typical intake of artificial sweeteners (in the range of milligrams per kilogram of body weight) is generally lower than the concentrations predicted by our models to be required for inducing significant biochemical perturbations. Future studies should integrate dose–response analysis, physiologically based pharmacokinetic (PBPK) modeling, and experimental validation to assess the biological significance under real-world exposure conditions. We hope these findings will advance further investigation into the roles of these core targets in sweetener-related musculoskeletal disease pathogenesis. This study has several limitations. First, although we predicted sweetener-target interactions through bioinformatics analysis and molecular docking, and performed preliminary molecular dynamics simulations for validation, these computational results have not been directly validated in cellular experiments, animal models, or clinical observations [42]. Whether sweeteners indeed act through these targets *in vivo* and initiate disease via the predicted networks requires further experimental evidence. Second, this study focused on “shared” mechanisms across musculoskeletal disorders, which is crucial for understanding common risks. However, different diseases (such as arthritis versus osteoporosis) may possess distinct key pathways and specific targets. The current shared target map may not fully capture these disease-specific mechanisms. Third, our research focused on a set of core shared targets, providing a clear framework. However, the human body is a complex system. The actual impact of sweeteners may involve broader gene interactions, epigenetic regulation, and systemic changes such as gut microbiota modulation, which were not incorporated into our core analytical network [43,44]. To address these limitations, future research can be pursued in the following directions. At the basic research level, experimental validation is recommended to establish causal relationships. *In vivo* animal experiments and *in vitro* cell studies should be conducted to directly verify the functional necessity of key targets in sweetener-induced musculoskeletal pathology. Additionally, the biological effects of sweeteners depend not only on the parent compounds but also significantly on their metabolic fate and gut microbiota-mediated transformation. Based on their interaction patterns with the gut microbiota, sweeteners can be classified into three categories. Saccharin and sucralose are minimally absorbed and reach the colon largely intact, where they directly modulate microbial structure and metabolism (e.g., altering short-chain fatty acid production and promoting antibiotic resistance gene transfer); they are thus defined as “microbiota-modulating” sweeteners. Steviol glycosides require hydrolysis by the gut microbiota into the aglycone steviol before absorption into the systemic circulation, making them “microbiota-dependent” sweeteners; consequently, the blind docking binding potential of their parent molecules does not represent the actual activity of the bioactive metabolites. Aspartame, in contrast, is mainly metabolized by host intestinal and hepatic enzyme systems into its constituent amino acids and methanol, with limited direct interaction with the gut microbiota, and therefore belongs to the “limited-interaction” type. These microbiota-mediated mechanisms cannot be captured by direct molecular docking between sweeteners and host proteins, and the exposure levels required to elicit significant physiological effects are often higher than realistic intake levels. Therefore, although blind docking has systematically identified potential allosteric sites and novel binding pockets of parent sweeteners with target proteins, these theoretical binding potentials must be interpreted with caution, taking into account metabolic pathways, microbiota-mediated transformation, and the principle that “the dose makes the poison”. Future studies integrating metabolomics, bioactive metabolite identification, and functional assessment of the gut microbiota are needed to validate their physiological relevance. This would enable unbiased discovery of novel key pathways and interactions, providing a more comprehensive understanding of their complex biological effects. Furthermore, stratified modeling or analysis can be performed for different diseases such as osteoporosis, osteoarthritis, and sarcopenia. This would help distinguish common versus specific pathways affected by sweeteners, offering mechanistic insights for precision prevention. At the public health and policy level, multidimensional interventions should be advocated. The public should be encouraged to improve metabolic health through regular physical activity and weight management. Research and development of safer sugar substitutes or natural sweetener alternatives should be promoted. It is also recommended that guidelines and standards for sweetener use be established or refined based on health risk assessments, aiming to reduce the long-term musculoskeletal burden associated with sweetener exposure.

## Conclusion

This study employed network toxicology and molecular docking to systematically investigate the potential molecular mechanisms by which sweetener exposure affects the musculoskeletal system, including intervertebral disc degeneration, myositis, osteoarthritis, and osteoporosis. Key candidate targets and related dysregulated pathways were identified. These findings provide a theoretical basis for understanding the musculoskeletal toxicity of sweeteners. This work establishes a computational biology foundation for subsequent mechanistic exploration. Future studies should experimentally validate the interactions between targets and pathways. This will facilitate the development of risk assessment and intervention strategies.

## Supporting information

S1 File Comprehensive supporting data for the study. This zip archive contains five folders: (1) Target information – target names, IDs, and sequences; (2) PPI network – interaction data and network files; (3) GO and KEGG results – enrichment tables and statistics. Refer to the Methods section for detailed descriptions of each analysis. (4) Molecular docking – input/output files and docking scores; (5) Molecular dynamics – trajectory files and simulation analysis results.
(ZIP)
