# Supplementary material for: A report on the mechanisms of sweeteners in four musculoskeletal disorders: Insights from network toxicology, molecular docking, and molecular dynamics simulations
Source: PLoS One. 2026 Aug 11;21(8):e0347282. doi: 10.1371/journal.pone.0347282 (PMC13460625; doi:10.1371/journal.pone.0347282)
Supplement: Supplementary file 1 [file pone.0347282.s002.docx]

**Dear Editor and Reviewers,**

Thank you very much for your valuable time and constructive comments on our manuscript. We greatly appreciate your recognition of the study’s potential as well as the insightful suggestions for improvement. We have carefully considered all the comments and revised the manuscript accordingly. The point-by-point responses are listed below, with all changes highlighted in the revised manuscript (highlight in yellow).

Editor’s Comments:

 When submitting your revision, we need you to address these additional requirements.

1. Please ensure that your manuscript meets PLOS ONE's style requirements, including those for file naming. The PLOS ONE style templates can be found at [https://journals.plos.org/plosone/s/file?id=wjVg/PLOSOne_formatting_sample_main_body.pdf](https://track.editorialmanager.com/CL0/https://journals.plos.org/plosone/s/file?id=wjVg/PLOSOne_formatting_sample_main_body.pdf/1/010f019e14c86a15-2540c9bf-8fdb-4c79-8022-4d07bbb270e1-000000/YE6vhPGyQuHXrTCFHTLFOmkaF3235JWWitJQQD1pXfU=258) and [https://journals.plos.org/plosone/s/file?id=ba62/PLOSOne_formatting_sample_title_authors_affiliations.pdf](https://track.editorialmanager.com/CL0/https://journals.plos.org/plosone/s/file?id=ba62/PLOSOne_formatting_sample_title_authors_affiliations.pdf/1/010f019e14c86a15-2540c9bf-8fdb-4c79-8022-4d07bbb270e1-000000/P-jt1fFajc3J1Uw07dTY4sbFFOMTI264dcURmCBYHqQ=258)

2. Please note that PLOS One has specific guidelines on code sharing for submissions in which author-generated code underpins the findings in the manuscript. In these cases, we expect all author-generated code to be made available without restrictions upon publication of the work. Please review our guidelines at https://journals.plos.org/plosone/s/materials-and-software-sharing#loc-sharing-code and ensure that your code is shared in a way that follows best practice and facilitates reproducibility and reuse.

3. Thank you for stating the following financial disclosure:"This work was supported by the Ningxia Natural Science Foundation Project (grant 2023AAC03543)" .Please state what role the funders took in the study.  If the funders had no role, please state: "The funders had no role in study design, data collection and analysis, decision to publish, or preparation of the manuscript."If this statement is not correct you must amend it as needed.Please include this amended Role of Funder statement in your cover letter; we will change the online submission form on your behalf.

4. Please include captions for your Supporting Information files at the end of your manuscript, and update any in-text citations to match accordingly. Please see our Supporting Information guidelines for more information: [http://journals.plos.org/plosone/s/supporting-information](http://track.editorialmanager.com/CL0/http://journals.plos.org/plosone/s/supporting-information/1/010f019e14c86a15-2540c9bf-8fdb-4c79-8022-4d07bbb270e1-000000/SHLQJy3au0xwnVGRTkVfu-KYBcYjN9CGHF2X_Y8UZBc=258).

Response: Thank you for your detailed instructions regarding the revision of our manuscript. We have addressed each point as follows:

1.Formatting and file naming: We have carefully reviewed the PLOS ONE style templates and have reformatted our manuscript accordingly, including the title page, main text, and figure file naming conventions.

2.Code sharing: We greatly appreciate your concern regarding the reproducibility of our work. The suggestion to share code is very important. However, in our study, all analyses were performed using the standard functions of established software and websites, and no custom scripts (code) were written or used. All software used has been described in the Materials and Methods section of the manuscript.

3.Role of funder statement: Upon verification, the funder of this study (Ningxia Natural Science Foundation, grant number 2023AAC03543) provided only financial support and had no involvement or influence in the study design, data collection and analysis, decision to publish, or preparation of the manuscript. Therefore, the following statement has been added to the revised cover letter:

“The funders had no role in study design, data collection and analysis, decision to publish, or preparation of the manuscript.”

4.Supporting Information captions: We have now added complete captions for all Supporting Information files at the end of the manuscript (after the References section) and have verified that all in‑text citations to these files match the captions.

Please do not hesitate to contact us if any further changes are required.

Reviewer #1:

Comment 1: Since the work emphasizes binding affinity, a table to show the result of the binding affinities and their corresponding values would improve the result session.

Response:Thank you for this constructive suggestion. We agree that a clear presentation of binding affinity values is very important for the results section. Following the reviewer’s advice, we have now presented the binding affinity values in Fig. 5, which summarizes all binding affinities and their corresponding values. We believe this revision significantly improves the clarity and persuasiveness of our results.

Comment 2: Toxicological relevance and dose considerations

The interpretation of the docking and simulation results should be framed within the fundamental toxicological principle that “the dose makes the poison.” While insilico approaches are valuable for predicting potential molecular interactions, they do not inherently reflect physiological exposure conditions. Importantly, artificial sweeteners are typically consumed at relatively low concentrations and are used in amounts often below those required to drive significant biochemical perturbations in vivo. Additionally, unlike reducing sugars, they are less likely to participate in non-enzymatic glycation reactions and the formation of advanced glycation end products (AGEs).The authors should therefore contextualize their findings within realistic exposure levels, clearly distinguishing between theoretical binding potential and biological risk.

Response:Thank you very much for this important and insightful comment. We fully agree with the reviewer that toxicological conclusions must be grounded in the principle that “the dose makes the poison,” and that our in silico results should be interpreted with careful consideration of real‑world exposure levels. In response to this valuable suggestion, we have added a new paragraph in the Discussion section to explicitly address the dose‑relevance of our findings. In this paragraph, we acknowledge that although our docking and simulation results suggest potential molecular interactions, the actual in vivo significance is contingent upon the achievable local and systemic concentrations of artificial sweeteners under typical consumption patterns. We cite published data indicating that these concentrations are generally low (often in the micromolar or sub‑micromolar range) and are unlikely to drive the strong biochemical perturbations suggested by the raw binding affinity values.These changes can be found on Page 21, Lines 585–591 (the newly added paragraph in the Discussion section). Once again, we thank the reviewer for guiding us to improve the scientific rigor of our manuscript.

Comment 3: Integration of metabolism and microbiota (key mechanistic gap)

The manuscript does not consider or mention anything about the metabolism of the investigated sweeteners, which is critical for accurate toxicological and physiological interpretation. The biological effects of these compounds are often determined not only by the parent molecules but also by their metabolic fate and biotransformation products.Furthermore, emerging evidence suggests that certain artificial sweeteners may influence host metabolism indirectly through modulation of gut microbiota composition and function. Such alterations can affect key metabolic pathways, including short-chain fatty acid production, glucose homeostasis, and inflammatory signaling.A more comprehensive discussion integrating metabolic pathways of the sweeteners, potential bioactive metabolites, and microbiota-mediated mechanisms would substantially enhance the physiological relevance of the study and provide a more balanced interpretation of the simulation results.

Response:Thank you very much for pointing out this critical mechanistic gap. We fully agree with the reviewer that a comprehensive toxicological and physiological interpretation requires consideration of both the metabolism of the parent sweeteners and the potential modulatory effects of the gut microbiota. In response to this valuable suggestion, we have made the following revisions to the manuscript: We have added a new paragraph in the Discussion section that explicitly addresses the metabolic fate of the investigated sweeteners. We now discuss that many of these compounds are poorly metabolized in humans but may undergo limited biotransformation in the gut or liver. We also discuss that artificial sweeteners can alter the composition and function of the gut microbiota, leading to changes in short‑chain fatty acid production, glucose homeostasis, and inflammatory signaling. Furthermore, we state that future studies should integrate metabolic profiling and microbiota analysis to translate our computational predictions into a more complete risk assessment framework. These changes can be found on Page 21, Lines 568–585 (the newly added paragraph in the Discussion section). Once again, we thank the reviewer for guiding us to fill this key mechanistic gap.

Reviewer #2:

Comment 1: Transparency in the screening of targets for network pharmacology needs to be improved

The abstract and main text do not specify the source database for sweetener targets, the screening thresholds, or the criteria used to define the 35 “core targets.” The transparency of this process directly affects the reproducibility of the results. It is recommended to provide a detailed list of the databases used and the screening workflow, and to include a brief justification or sensitivity analysis for key thresholds to strengthen the basis of the predictions.

Response:Thank you for raising this important issue regarding transparency and reproducibility. We fully agree that the screening process for network pharmacology targets must be clearly documented. In our original manuscript, we did not provide sufficient detail on the database sources, screening thresholds, or the criteria used to define the 35 core targets. We have now thoroughly revised this section. Specifically, we have described in the Methods section all the databases used to retrieve sweetener‑related targets, and we have provided a detailed description of the screening process for the core targets (Page 4, Lines 94–105). We thank the reviewer for guiding us to improve the transparency and reproducibility of our analysis.

Comment 2: Presentation of molecular docking results should avoid selective bias

The abstract repeatedly emphasizes the prominent binding potential of luo han guo glycosides and stevioside, yet fails to disclose the total number of tested sweeteners and the distribution of docking scores, which may create the misleading impression that “only these two sweeteners pose a risk.” It is recommended to use a supplementary table to comprehensively list the binding energies of each sweetener with all core targets and to objectively rank risks based on a unified docking score threshold.

Response:Thank you for this valuable and constructive comment. We fully agree that selectively presenting docking results could lead to bias, and we apologize for any misleading impression caused by the original wording. To address this issue and improve the transparency and objectivity of our analysis, we have now listed the binding energies of each sweetener with all core targets in Fig. 5. This table allows readers to view the complete distribution of docking scores and to compare across different sweetener–target pairs. We believe these revisions fully address the concern regarding selective bias and provide a transparent, comprehensive, and objective presentation of the docking results. We thank the reviewer for guiding us to improve the scientific integrity of our manuscript.

Comment 3: Key methodological details of molecular dynamics simulations must be supplemented

The reliability of the conclusions regarding “binding stability” is highly dependent on simulation parameters. Please provide the simulation duration, force field, temperature and pressure conditions, number of independent repetitions, and stability assessment metrics. If necessary, provide RMSD/RMSF plots to fully support the conclusions regarding binding stability with FASN, NOS2, and PCSK9.

Response:Thank you for pointing out the need to provide more detailed methodological information for the molecular dynamics simulations. We fully agree that the reliability of the binding stability conclusions depends on transparent reporting of simulation parameters. In the original manuscript, these details were insufficiently described. We have now provided RMSD/RMSF plots in Fig. 7 to support the binding stability conclusions for FASN, NOS2, and PCSK9. We believe these additions fully address the reviewer’s concerns and provide a solid basis for our conclusions on binding stability.

Comment 4: Incorporate the in vivo metabolism of sweeteners and tissue exposure concentrations into the discussionMolecular docking is based on parent molecules, whereas some sweeteners (e.g., stevioside) undergo significant metabolic transformation in vivo, and actual exposure concentrations in bone and joint tissues are far lower than those in the simulated system. It is recommended to use predictive ADME tools to estimate metabolite and tissue distribution, or to clearly define the physiological concentration assumptions underlying the current “binding potential” in the discussion, thereby making the conclusions more cautious.

Response:Thank you for raising this critical question regarding the in vivo relevance of our docking results. We fully agree that molecular docking based solely on parent molecules, without consideration of metabolic transformation and actual tissue exposure concentrations, may overestimate physiological significance. This is an important limitation that we have now addressed in the revised manuscript. Specifically, we have added a new paragraph in the Discussion section stating that toxicological conclusions must be grounded in the principle that “the dose makes the poison,” and that our in silico results should be interpreted with careful consideration of realistic exposure levels. We also specifically note that actual exposure concentrations in bone and joint tissues are likely far lower than the concentrations used in our simulations, further reducing the physiological relevance of the raw binding scores.These changes can be found on Page 21, Lines 585–591 (the newly added paragraph in the Discussion section). Once again, we thank the reviewer for guiding us to improve the scientific rigor of our manuscript.

Comment 5: Specific evidence is required to establish the association between core targets and musculoskeletal tissues.Inflammatory responses and metabolic disorders are broad pathways and are insufficient to establish specific links to diseases such as intervertebral disc degeneration and myositis. It is recommended to integrate tissue expression profile data from sources such as GTEx to elucidate the expression levels and functional weights of the 35 core targets in tissues such as bone, cartilage, and muscle, thereby strengthening the disease relevance of these targets.

Response:We acknowledge that tissue‑specific expression data (e.g., from GTEx) would strengthen the association. While we have not performed such analysis in the current study due to resource limitations, we have added a limitation in the Discussion stating that future studies should incorporate tissue expression profiling to validate target relevance in bone/cartilage/muscle.

Comment 6: Network analysis of the four diseases should be conducted using a stratified approach

Although intervertebral disc degeneration, myositis, osteoarthritis, and osteoporosis all fall under the musculoskeletal category, they exhibit distinct molecular pathological differences. Combined analysis may dilute disease-specific signals. It is recommended to supplement the analysis with separate enrichment results for each disease or to employ disease-specific network comparisons to reveal potential differential patterns of sweetener effects across different diseases.

Response:Thank you for this insightful and methodologically important comment. We fully agree that although the four diseases (intervertebral disc degeneration, myositis, osteoarthritis, and osteoporosis) all fall under the musculoskeletal category, they exhibit distinct molecular pathological differences. Using the intersection of sweetener‑related targets with the disease‑specific target set for each disease, we performed independent GO and KEGG enrichment analyses for each disease. These results are presented in Fig. 1, Fig. 2, Fig. 3, and Fig. 4. We thank the reviewer for guiding us to adopt a more refined, disease‑stratified analytical strategy, which has substantially strengthened the specificity and translational value of our study.

Comment 7: The discussion section should include a cross-comparison with existing experimental toxicological evidence.The current discussion directly links computational findings to subsequent experimental validation but fails to engage with published cellular/animal studies or clinical observations on sweeteners. It is recommended to compare data on inflammation, metabolism, and bone health from existing experimental literature to analyze the consistency between computational predictions and actual reports, which will significantly enhance the biological credibility of the computational results.

Response:Thank you for this valuable suggestion. We agree that comparing our computational predictions with existing experimental toxicological evidence (cellular/animal studies and clinical observations) is essential to enhance the biological credibility of our findings. In the original manuscript, this cross‑comparison was insufficient. We have now substantially revised the Discussion section to address this gap. Specifically, we have added a new paragraph stating that although the magnitude of effects in vivo strongly depends on dose and exposure duration, our docking results are broadly consistent with these reports. These additions can be found on Page 21, Lines 568–591.

Comment 8: The conclusion section should clearly define the limitations and uncertainties of purely computational studies.While emphasizing “new targets and theoretical foundations,” the conclusion should clearly state that the current evidence is purely computational inference and lacks key data such as dose-response relationships, tissue exposure concentrations, and in vivo dynamic regulation. Including this qualification not only does not detract from the study’s innovativeness but also makes the research stance more scientific and rigorous.

Response:Thank you for this important comment. We fully agree that while emphasizing “new targets and theoretical foundations,” the conclusion should clearly state that the current evidence is purely computational inference and lacks key data such as dose‑response relationships, tissue exposure concentrations, and in vivo dynamic regulation. We have revised the Conclusion section accordingly. These modifications can be found on Page 18, Lines 534–547. We believe that including this qualification does not detract from the study’s innovativeness but rather makes our research stance more scientific and rigorous.

Reviewer #3:

Comment:This work investigates the links between sweeteners and musculoskeletal diseases by leveraging publicly available data from reliable sources that are well established within the scientific community. Network metrics were used to identify key genes, which were then subjected to enrichment analysis to provide insights into the potential underlying mechanisms. Molecular docking was subsequently performed to investigate interactions between sweeteners and candidate gene products. This work highlights several targets of interest that may guide further experimental investigations.

A minor issue with the manuscript is that the font size of labels in all figures is too small to be easily readable. As a result, the evaluation of the results relied primarily on the tables provided in the supplementary materials.

In my opinion, the major issue with the manuscript is insufficient methodological reporting. While the Materials and Methods section mentions the tools used, it provides very limited detail regarding parameter choices, and these are neither justified nor fully described. Additionally, the underlying statistical tests and methodological principles of the tools are not specified. Information such as tool versions and database access dates is also missing. Altogether, this lack of detail makes the findings difficult to reproduce and evaluate, and potentially impossible to replicate in the case of tool discontinuation or substantial updates.

Regarding the methodology, a key concern is that pathway interpretation mainly relies on top hits ranked by p-value. This approach does not account for effect size and may inflate the importance of biologically irrelevant findings. In some datasets, p-value filtering followed by z-score ranking highlights pathways and functions that are not discussed (such as infection-related processes), raising concerns about the consistency of the interpretation. I would recommend revisiting the enrichment analysis and its interpretation with appropriate consideration of effect size.

Response:We sincerely thank the reviewer for the thorough and constructive assessment of our manuscript. We appreciate the reviewer’s positive recognition of our overall approach and the clear identification of specific areas that need improvement. We have carefully addressed each point as detailed below.

1.Figure label font sizes: We agree that the font size of labels in all figures is too small to be easily readable. To resolve this, we have increased the font size of all annotations in all figures. The revised figures have been uploaded with the updated manuscript. We believe the improved readability will allow readers to evaluate the results without relying solely on the supplementary tables.

2.Methodological reporting: We fully agree that insufficient methodological detail compromises reproducibility. We have expanded the Materials and Methods section to clearly state the default or chosen parameters for each tool, along with brief justifications. All software versions are now presented in the Methods section. These revisions can be found on Page 4, Lines 94–141.

3.Enrichment analysis and effect size: We have re‑evaluated the top‑ranked pathways and now focus on those that are both statistically significant (adjusted p < 0.05) and show a substantial enrichment ratio (> 2.0 or appropriate threshold), specifically the top 10 pathways. The corresponding table has been uploaded as supplementary material. Pathways with high statistical significance but weak enrichment (e.g., infection‑related terms) are explicitly acknowledged as likely artifacts and are no longer emphasized in the main discussion.

Once again, we thank the reviewer for the rigorous and helpful feedback, which has significantly improved the quality of our manuscript.

Reviewer #4:

Comment 1: Congratulations. You have done an interesting job constructing a comprehensive computational pipeline to explore a highly relevant public health question. The integration of network toxicology with molecular dynamics simulations provides a solid theoretical foundation for understanding how sweeteners might influence musculoskeletal diseases. The manuscript is clearly written, and your transparency regarding the study limitations in the discussion section well addressed.

Here are a few minor suggestions to further strengthen your manuscript before publication:

Clarification on Blind Docking. You mention using a "blind docking approach to conduct an unbiased exploration of binding sites" for the 350 sweetener-protein pairs. While this is a valid exploratory strategy, blind docking can sometimes lack the precision of targeted docking, especially for large proteins like FASN. Please expand in the discussion on why this approach was chosen over targeted docking and acknowledge it as a methodological limitation as well.

The binding energies presented in Figure 5 are insightful, but it would be helpful to provide a brief comparative baseline in the text. Discuss what constitutes a "strong" binding energy threshold in AutoDock Vina to help readers who may not be experts in computational chemistry immediately identify the significance of your results.

Discussion of genes and its functions. The name of the genes/proteins should be described completely in this section while describing its function, and not only mention as an acronyms/shorten name.

Figure Presentation. In the draft PDF, your figures (especially the molecular dynamics plots in Figure 7) are dense with valuable data but the resolution in not good. The readers will require to zoom in to interpret the graphs, so ensure it is clear enough for a significant zoom in.

Minor text edits. There are several phrases that lacks of a space after the ending point. For example, page 12, in the middle of sections 2.4: "....are available in the Supplementary File.In this large-scale screening...,". Please re-review all the draft to ensure this consistent error is corrected.

Italics are missing in all the draft text, for the species naming and other terms naming (i.e. "homo sapiens", "in vivo", "in vitro"). Please re-review all the draft to ensure this consistent error is corrected.

Response:We sincerely thank the reviewer for the positive and constructive feedback on our manuscript. We are glad that our computational pipeline and integration of network toxicology with molecular dynamics simulations are considered a solid theoretical foundation for addressing a relevant public health question. We also appreciate the reviewer’s acknowledgment of our transparent discussion of limitations in the Discussion section. Below we address each of the minor suggestions point‑by‑point.

1.Blind docking clarification: Thank you for this important methodological suggestion. We agree that the precision of blind docking versus targeted docking should be discussed. In the revised Discussion section, we have added a new paragraph explaining that a blind docking approach was chosen because no prior structural information on sweetener binding sites for most of the 350 targets was available, and we aimed to perform an unbiased exploratory screening. However, we now explicitly acknowledge that blind docking may reduce precision for large, multi‑domain proteins such as FASN, and that this constitutes a methodological limitation. We also suggest that future targeted docking or experimental structure determination could validate the predicted binding modes. This addition can be found on Page 17, Lines 506–509.

2.Binding energy threshold: Thank you for this helpful suggestion. We have added a brief explanation that in AutoDock Vina, binding energies (affinities) are typically reported in kcal/mol. Based on established literature, we define ≤ –7.0 kcal/mol as strong binding, and this threshold is now cited with appropriate references. See Page 18, Lines 511–513.

3.Full gene/protein names: We completely agree. We have carefully reviewed the entire manuscript and revised all sections to ensure that each gene or protein is introduced with its full name followed by the accepted acronym in parentheses. We have added this baseline information so that non‑expert readers can immediately understand the significance of our results. See Page 16, Lines 440–503.

4.Figure resolution: We apologize for the poor resolution in the draft PDF. We have now re‑generated Figure 7 (and all other figures with similar issues) at 600 dpi in vector format (PDF/TIFF). Even at high zoom levels, the labels, axes, and curves are sharp and clear. The revised high‑resolution figures have been uploaded with the manuscript. We confirm that the updated figure files are suitable for publication.

5.Minor text edits (missing spaces after periods): We thank the reviewer for catching this typographical error. We have performed a complete copy‑edit of the entire manuscript to ensure that every period is followed by a single space.

6.Missing italics: We apologize for this oversight. We have now systematically italicized all scientific species names (e.g., Homo sapiens, Mus musculus, Escherichia coli) and standard Latin terms (e.g., in vivo, in vitro, in silico, ex vivo) throughout the manuscript. This correction has been applied to the main text, figure legends, and supplementary materials.

We thank the reviewer for these valuable suggestions, which have significantly improved the clarity, reproducibility, and professional presentation of our work.

Sincerely,

Haifeng Yuan, on behalf of all authors
