# Supplementary material for: A report on the mechanisms of sweeteners in four musculoskeletal disorders: Insights from network toxicology, molecular docking, and molecular dynamics simulations
Source: PLoS One. 2026 Aug 11;21(8):e0347282. doi: 10.1371/journal.pone.0347282 (PMC13460625; doi:10.1371/journal.pone.0347282)
Supplement: Supplementary file 3 [file pone.0347282.s003.docx]

**Dear Editor and Reviewers,**

We sincerely thank the editor and the reviewers for their time and constructive comments on our manuscript. We have carefully addressed each point raised, and the detailed revisions are outlined below. All changes in the revised manuscript have been highlighted using the "Track Changes" mode in Microsoft Word for easy reference. We believe these revisions have substantially improved the quality of our work, and we hope the manuscript now meets the standards for publication in PLOS ONE.

**Response to Reviewer #2:**

Reviewer’s comment:This manuscript delivers solid and meaningful research progress in its corresponding research domain, with a well-defined and innovative research topic that effectively fills existing research gaps in this field. The authors systematically sorted out relevant previous literature, clearly illustrated the research background, research objectives and expected research contributions at the beginning of the article. The whole experimental scheme is reasonable and complete, multiple validated experimental techniques were applied to support the core research conclusion, and all original data and supplementary figures are sufficient and credible. The discussion section thoroughly interprets the experimental findings, objectively analyzes the limitations of this study, and puts forward feasible directions for subsequent follow-up research. The manuscript has clear chapter logic, standardized figure formatting and fluent academic writing, with only trivial grammatical expression and reference format issues to adjust. No serious scientific defects, ethical risks or repeated publication problems are found in this work. Overall, this paper satisfies the publication criteria of this journal, and I strongly recommend accepting this manuscript after minor revisions.

Response:We sincerely thank the reviewer for the highly positive and encouraging evaluation of our work. The reviewer's recognition that our research topic is innovative, the experimental design is reasonable, the data are sufficient, and the discussion is thorough is greatly appreciated. We also take seriously the reviewer’s suggestions regarding minor grammatical expression and reference formatting issues. In this revision, we have carefully copy-edited the entire manuscript to improve language accuracy and fluency, and we have meticulously checked and corrected all references to conform to the journal’s formatting requirements.We are deeply grateful for the reviewer’s time and valuable guidance.

**Response to Reviewer #4：**Reviewer’s comment:Thanks for the work on addressing the comments from the last round of review, especially on the methodology and discussion sections. Even though the quality of the draft has improved substantially, there are still some minor text suggestions to improve the written quality of the manuscript before publication, most of which are carried from comments not addressed in the previous review comments:

- Minor text edits. There are still several phrases that lack a space after the period in the text (some examples in lines 126, 156, 172,183, 189, and so on...). Please re-review ALL the draft text thoroughly and carefully to ensure this consistent error is corrected in the text, figure captions, and supplemental material.

- Line 56. "Notably, Some existing studies on....". Correct the uppercase "S" in "some".

- Italics are still missing in the CLEAN draft text for the species naming and other term naming (i.e., "homo sapiens", "mus musculus", "in vivo", "in vitro", etc.). Nevertheless, in the markdown version, this error seems to be corrected, but not consistently. Please re-review ALL the draft text thoroughly and carefully to ensure this is corrected in the draft text, figure captions, and supplemental material.

- p-value should be named with lowercase “p”. Nevertheless, there are several “P-value” mentions that need to be corrected to “p-value” throughout the text. Please re-review ALL the draft text thoroughly and carefully to ensure this issue is corrected in the draft text, figure captions AND supplemental material texts.

Your work will increase its quality after these minor edits are implemented. Best of luck with the next stages of publication.

Response:We sincerely thank the reviewer for taking the time to re-evaluate our manuscript and for carefully pointing out these detailed textual issues. We highly value these suggestions and have addressed each one as follows:

1. Regarding the missing spaces after periods: We have thoroughly and meticulously reviewed the entire manuscript, including all figure captions and supplemental material, and have corrected all instances where a space was missing after a period (including at lines 126, 156, 172, 183, 189, and elsewhere as noted by the reviewer).
2. Regarding the uppercase "S" in "Some" at Line 56: We have corrected this to lowercase "some". The sentence now reads: "Notably, some existing studies on...".
3. Regarding the missing italics for species names and Latin terms: We have systematically reviewed the entire manuscript, all figure captions, and supplemental material to ensure that all species names (e.g., Homo sapiens, Mus musculus) and Latin terms (e.g., in vivo, in vitro) are now consistently italicized throughout.
4. Regarding the capitalization of "p-value": We have uniformly corrected all instances of "P-value" to "p-value" throughout the main text, figure captions, and supplemental material.

We are truly grateful for the reviewer's meticulous reading and valuable suggestions. We believe these revisions have further enhanced the written quality of our manuscript.

Sincerely,

Haifeng Yuan, on behalf of all authors
