# Supplementary material for: A report on the mechanisms of sweeteners in four musculoskeletal disorders: Insights from network toxicology, molecular docking, and molecular dynamics simulations
Source: PLoS One. 2026 Aug 11;21(8):e0347282. doi: 10.1371/journal.pone.0347282 (PMC13460625; doi:10.1371/journal.pone.0347282)
Supplement: Peer review history

# Peer review: Decision Letter 0

**DOI:** 10.1371/journal.pone.0347282.r001

**Authors:** Laxit K Bhatt

10 May 2026

Dear Dr. Yuan,

Thank you for submitting your manuscript to PLOS ONE. After careful consideration, we feel that it has merit but does not fully meet PLOS ONE’s publication criteria as it currently stands. Therefore, we invite you to submit a revised version of the manuscript that addresses the points raised during the review process.

Please submit your revised manuscript by Jun 24 2026 11:59PM. If you will need more time than this to complete your revisions, please reply to this message or contact the journal office at plosone@plos.org. When you're ready to submit your revision, log on to https://www.editorialmanager.com/pone/ and select the 'Submissions Needing Revision' folder to locate your manuscript file.

- A letter that responds to each point raised by the academic editor and reviewer(s). You should upload this letter as a separate file labeled 'Response to Reviewers'.
- A marked-up copy of your manuscript that highlights changes made to the original version. You should upload this as a separate file labeled 'Revised Manuscript with Track Changes'.
- An unmarked version of your revised paper without tracked changes. You should upload this as a separate file labeled 'Manuscript'.

If you would like to make changes to your financial disclosure, please include your updated statement in your cover letter. Guidelines for resubmitting your figure files are available below the reviewer comments at the end of this letter.

If applicable, we recommend that you deposit your laboratory protocols in protocols.io to enhance the reproducibility of your results. Protocols.io assigns your protocol its own identifier (DOI) so that it can be cited independently in the future. For instructions see: https://journals.plos.org/plosone/s/submission-guidelines#loc-laboratory-protocols. Additionally, PLOS ONE offers an option for publishing peer-reviewed Lab Protocol articles, which describe protocols hosted on protocols.io. Read more information on sharing protocols at https://plos.org/protocols?utm_medium=editorial-email&utm_source=authorletters&utm_campaign=protocols.

As the corresponding author, your ORCID iD is verified in the submission system and will appear in the published article. PLOS supports the use of ORCID, and we encourage all coauthors to register for an ORCID iD and use it as well. Please encourage your coauthors to verify their ORCID iD within the submission system before final acceptance, as unverified ORCID iDs will not appear in the published article. *Only*  the individual author can complete the verification step; PLOS staff *cannot*  verify ORCID iDs on behalf of authors.

We look forward to receiving your revised manuscript.

Kind regards,

Laxit K Bhatt

Academic Editor

PLOS One

Journal Requirements:

When submitting your revision, we need you to address these additional requirements.

1. Please ensure that your manuscript meets PLOS ONE's style requirements, including those for file naming. The PLOS ONE style templates can be found at

https://journals.plos.org/plosone/s/file?id=wjVg/PLOSOne_formatting_sample_main_body.pdf and

https://journals.plos.org/plosone/s/file?id=ba62/PLOSOne_formatting_sample_title_authors_affiliations.pdf

<h2>2. Please note that PLOS One has specific guidelines on code sharing for submissions in which author-generated code underpins the findings in the manuscript. In these cases, we expect all author-generated code to be made available without restrictions upon publication of the work. Please review our guidelines at https://journals.plos.org/plosone/s/materials-and-software-sharing#loc-sharing-code and ensure that your code is shared in a way that follows best practice and facilitates reproducibility and reuse.</h2><h2>3. Thank you for stating the following financial disclosure:</h2><h2>"This work was supported by the Ningxia Natural Science Foundation Project (grant 2023AAC03543)"        </h2><h2>Please state what role the funders took in the study.  If the funders had no role, please state: "The funders had no role in study design, data collection and analysis, decision to publish, or preparation of the manuscript."</h2><h2>If this statement is not correct you must amend it as needed.</h2><h2>Please include this amended Role of Funder statement in your cover letter; we will change the online submission form on your behalf.</h2>

4. Please include captions for your Supporting Information files at the end of your manuscript, and update any in-text citations to match accordingly. Please see our Supporting Information guidelines for more information: http://journals.plos.org/plosone/s/supporting-information.

If the reviewer comments include a recommendation to cite specific previously published works, please review and evaluate these publications to determine whether they are relevant and should be cited. There is no requirement to cite these works unless the editor has indicated otherwise.

[Note: HTML markup is below. Please do not edit.]

Reviewers' comments:

Reviewer's Responses to Questions

**Comments to the Author**

1. Is the manuscript technically sound, and do the data support the conclusions?

Reviewer #1: Partly

Reviewer #2: Yes

Reviewer #3: Partly

Reviewer #4: Yes

**********

2. Has the statistical analysis been performed appropriately and rigorously?

Reviewer #1: Yes

Reviewer #2: Yes

Reviewer #3: No

Reviewer #4: Yes

**********

3. Have the authors made all data underlying the findings in their manuscript fully available?

The PLOS Data policy

Reviewer #1: Yes

Reviewer #2: Yes

Reviewer #3: No

Reviewer #4: Yes

**********

4. Is the manuscript presented in an intelligible fashion and written in standard English?

Reviewer #1: Yes

Reviewer #2: Yes

Reviewer #3: Yes

Reviewer #4: Yes

**********

Reviewer #1: The rationale underlying the experimental design is sound, and the synthetic approach is clearly described and applicable. However, as is common with simulation-based studies, the acknowledged limitations render several conclusions speculative without sufficient biological contextualization. The following minor revisions are recommended:

1. Since the work emphasizes binding affinity, a table to show the result of the binding affinities and their corresponding values would improve the result session.

2. Toxicological relevance and dose considerations

The interpretation of the docking and simulation results should be framed within the fundamental toxicological principle that “the dose makes the poison.” While insilico approaches are valuable for predicting potential molecular interactions, they do not inherently reflect physiological exposure conditions. Importantly, artificial sweeteners are typically consumed at relatively low concentrations and are used in amounts often below those required to drive significant biochemical perturbations in vivo. Additionally, unlike reducing sugars, they are less likely to participate in non-enzymatic glycation reactions and the formation of advanced glycation end products (AGEs).

The authors should therefore contextualize their findings within realistic exposure levels, clearly distinguishing between theoretical binding potential and biological risk.

3. Integration of metabolism and microbiota (key mechanistic gap)

The manuscript does not consider or mention anything about the metabolism of the investigated sweeteners, which is critical for accurate toxicological and physiological interpretation. The biological effects of these compounds are often determined not only by the parent molecules but also by their metabolic fate and biotransformation products.

Furthermore, emerging evidence suggests that certain artificial sweeteners may influence host metabolism indirectly through modulation of gut microbiota composition and function. Such alterations can affect key metabolic pathways, including short-chain fatty acid production, glucose homeostasis, and inflammatory signaling.

A more comprehensive discussion integrating metabolic pathways of the sweeteners, potential bioactive metabolites, and microbiota-mediated mechanisms would substantially enhance the physiological relevance of the study and provide a more balanced interpretation of the simulation results.

Reviewer #2: This study features a novel research topic and a logically coherent technical approach, forming a well-integrated workflow from network toxicology through molecular docking to molecular dynamics simulations, thereby providing valuable theoretical predictions regarding the musculoskeletal effects of sweeteners. The above comments primarily address methodological transparency, the comprehensiveness of results presentation, and the depth of biological reasoning, all of which are achievable revision requirements. We recommend that the authors address each point individually and make the necessary revisions; the manuscript will be accepted upon revision.

1. Transparency in the screening of targets for network pharmacology needs to be improved

The abstract and main text do not specify the source database for sweetener targets, the screening thresholds, or the criteria used to define the 35 “core targets.” The transparency of this process directly affects the reproducibility of the results. It is recommended to provide a detailed list of the databases used and the screening workflow, and to include a brief justification or sensitivity analysis for key thresholds to strengthen the basis of the predictions.

2. Presentation of molecular docking results should avoid selective bias

The abstract repeatedly emphasizes the prominent binding potential of luo han guo glycosides and stevioside, yet fails to disclose the total number of tested sweeteners and the distribution of docking scores, which may create the misleading impression that “only these two sweeteners pose a risk.” It is recommended to use a supplementary table to comprehensively list the binding energies of each sweetener with all core targets and to objectively rank risks based on a unified docking score threshold.

3. Key methodological details of molecular dynamics simulations must be supplemented

The reliability of the conclusions regarding “binding stability” is highly dependent on simulation parameters. Please provide the simulation duration, force field, temperature and pressure conditions, number of independent repetitions, and stability assessment metrics. If necessary, provide RMSD/RMSF plots to fully support the conclusions regarding binding stability with FASN, NOS2, and PCSK9.

4. Incorporate the in vivo metabolism of sweeteners and tissue exposure concentrations into the discussion

Molecular docking is based on parent molecules, whereas some sweeteners (e.g., stevioside) undergo significant metabolic transformation in vivo, and actual exposure concentrations in bone and joint tissues are far lower than those in the simulated system. It is recommended to use predictive ADME tools to estimate metabolite and tissue distribution, or to clearly define the physiological concentration assumptions underlying the current “binding potential” in the discussion, thereby making the conclusions more cautious.

5. Specific evidence is required to establish the association between core targets and musculoskeletal tissues.

Inflammatory responses and metabolic disorders are broad pathways and are insufficient to establish specific links to diseases such as intervertebral disc degeneration and myositis. It is recommended to integrate tissue expression profile data from sources such as GTEx to elucidate the expression levels and functional weights of the 35 core targets in tissues such as bone, cartilage, and muscle, thereby strengthening the disease relevance of these targets.

6. Network analysis of the four diseases should be conducted using a stratified approach

Although intervertebral disc degeneration, myositis, osteoarthritis, and osteoporosis all fall under the musculoskeletal category, they exhibit distinct molecular pathological differences. Combined analysis may dilute disease-specific signals. It is recommended to supplement the analysis with separate enrichment results for each disease or to employ disease-specific network comparisons to reveal potential differential patterns of sweetener effects across different diseases.

7. The discussion section should include a cross-comparison with existing experimental toxicological evidence

The current discussion directly links computational findings to subsequent experimental validation but fails to engage with published cellular/animal studies or clinical observations on sweeteners. It is recommended to compare data on inflammation, metabolism, and bone health from existing experimental literature to analyze the consistency between computational predictions and actual reports, which will significantly enhance the biological credibility of the computational results.

8. The conclusion section should clearly define the limitations and uncertainties of purely computational studies.

While emphasizing “new targets and theoretical foundations,” the conclusion should clearly state that the current evidence is purely computational inference and lacks key data such as dose-response relationships, tissue exposure concentrations, and in vivo dynamic regulation. Including this qualification not only does not detract from the study’s innovativeness but also makes the research stance more scientific and rigorous.

Reviewer #3: This work investigates the links between sweeteners and musculoskeletal diseases by leveraging publicly available data from reliable sources that are well established within the scientific community. Network metrics were used to identify key genes, which were then subjected to enrichment analysis to provide insights into the potential underlying mechanisms. Molecular docking was subsequently performed to investigate interactions between sweeteners and candidate gene products. This work highlights several targets of interest that may guide further experimental investigations.

A minor issue with the manuscript is that the font size of labels in all figures is too small to be easily readable. As a result, the evaluation of the results relied primarily on the tables provided in the supplementary materials.

In my opinion, the major issue with the manuscript is insufficient methodological reporting. While the Materials and Methods section mentions the tools used, it provides very limited detail regarding parameter choices, and these are neither justified nor fully described. Additionally, the underlying statistical tests and methodological principles of the tools are not specified. Information such as tool versions and database access dates is also missing. Altogether, this lack of detail makes the findings difficult to reproduce and evaluate, and potentially impossible to replicate in the case of tool discontinuation or substantial updates.

Regarding the methodology, a key concern is that pathway interpretation mainly relies on top hits ranked by p-value. This approach does not account for effect size and may inflate the importance of biologically irrelevant findings. In some datasets, p-value filtering followed by z-score ranking highlights pathways and functions that are not discussed (such as infection-related processes), raising concerns about the consistency of the interpretation. I would recommend revisiting the enrichment analysis and its interpretation with appropriate consideration of effect size.

Reviewer #4: Dear authors,

Congratulations. You have done an interesting job constructing a comprehensive computational pipeline to explore a highly relevant public health question. The integration of network toxicology with molecular dynamics simulations provides a solid theoretical foundation for understanding how sweeteners might influence musculoskeletal diseases. The manuscript is clearly written, and your transparency regarding the study limitations in the discussion section well addressed.

Here are a few minor suggestions to further strengthen your manuscript before publication:

- Clarification on Blind Docking. You mention using a "blind docking approach to conduct an unbiased exploration of binding sites" for the 350 sweetener-protein pairs. While this is a valid exploratory strategy, blind docking can sometimes lack the precision of targeted docking, especially for large proteins like FASN. Please expand in the discussion on why this approach was chosen over targeted docking and acknowledge it as a methodological limitation as well.

- The binding energies presented in Figure 5 are insightful, but it would be helpful to provide a brief comparative baseline in the text. Discuss what constitutes a "strong" binding energy threshold in AutoDock Vina to help readers who may not be experts in computational chemistry immediately identify the significance of your results.

- Discussion of genes and its functions. The name of the genes/proteins should be described completely in this section while describing its function, and not only mention as an acronyms/shorten name.

- Figure Presentation. In the draft PDF, your figures (especially the molecular dynamics plots in Figure 7) are dense with valuable data but the resolution in not good. The readers will require to zoom in to interpret the graphs, so ensure it is clear enough for a significant zoom in.

- Minor text edits. There are several phrases that lacks of a space after the ending point. For example, page 12, in the middle of sections 2.4: "....are available in the Supplementary File.In this large-scale

screening...,". Please re-review all the draft to ensure this consistent error is corrected.

- Italics are missing in all the draft text, for the species naming and other terms naming (i.e. "homo sapiens", "in vivo", "in vitro"). Please re-review all the draft to ensure this consistent error is corrected.

Your work provides a good springboard for future experimental validation. Best of luck with the next stages of publication.

**********

what does this mean?). If published, this will include your full peer review and any attached files.

If you choose “no”, your identity will remain anonymous but your review may still be made public.

**Do you want your identity to be public for this peer review?**  For information about this choice, including consent withdrawal, please see our Privacy Policy

Reviewer #1: No

Reviewer #2: No

Reviewer #3: No

Reviewer #4: **Yes**

**********

[NOTE: If reviewer comments were submitted as an attachment file, they will be attached to this email and accessible via the submission site. Please log into your account, locate the manuscript record, and check for the action link "View Attachments". If this link does not appear, there are no attachment files.]

To ensure your figures meet our technical requirements, please review our figure guidelines: https://journals.plos.org/plosone/s/figures

You may also use PLOS’s free figure tool, NAAS, to help you prepare publication quality figures: https://journals.plos.org/plosone/s/figures#loc-tools-for-figure-preparation.

NAAS will assess whether your figures meet our technical requirements by comparing each figure against our figure specifications.

# Author comment: Author response to Decision Letter 1

**DOI:** 10.1371/journal.pone.0347282.r002

23 May 2026

Dear Editor and Reviewers,

Thank you very much for your valuable time and constructive comments on our manuscript. We greatly appreciate your recognition of the study’s potential as well as the insightful suggestions for improvement. We have carefully considered all the comments and revised the manuscript accordingly. The point-by-point responses are listed below, with all changes highlighted in the revised manuscript (highlight in yellow).

Editor’s Comments:

When submitting your revision, we need you to address these additional requirements.

1. Please ensure that your manuscript meets PLOS ONE's style requirements, including those for file naming. The PLOS ONE style templates can be found at https://journals.plos.org/plosone/s/file?id=wjVg/PLOSOne_formatting_sample_main_body.pdf and https://journals.plos.org/plosone/s/file?id=ba62/PLOSOne_formatting_sample_title_authors_affiliations.pdf

2. Please note that PLOS One has specific guidelines on code sharing for submissions in which author-generated code underpins the findings in the manuscript. In these cases, we expect all author-generated code to be made available without restrictions upon publication of the work. Please review our guidelines at https://journals.plos.org/plosone/s/materials-and-software-sharing#loc-sharing-code and ensure that your code is shared in a way that follows best practice and facilitates reproducibility and reuse.

3. Thank you for stating the following financial disclosure:"This work was supported by the Ningxia Natural Science Foundation Project (grant 2023AAC03543)" .Please state what role the funders took in the study.  If the funders had no role, please state: "The funders had no role in study design, data collection and analysis, decision to publish, or preparation of the manuscript."If this statement is not correct you must amend it as needed.Please include this amended Role of Funder statement in your cover letter; we will change the online submission form on your behalf.

4. Please include captions for your Supporting Information files at the end of your manuscript, and update any in-text citations to match accordingly. Please see our Supporting Information guidelines for more information: http://journals.plos.org/plosone/s/supporting-information.

Response: Thank you for your detailed instructions regarding the revision of our manuscript. We have addressed each point as follows:

1.Formatting and file naming: We have carefully reviewed the PLOS ONE style templates and have reformatted our manuscript accordingly, including the title page, main text, and figure file naming conventions.

2.Code sharing: We greatly appreciate your concern regarding the reproducibility of our work. The suggestion to share code is very important. However, in our study, all analyses were performed using the standard functions of established software and websites, and no custom scripts (code) were written or used. All software used has been described in the Materials and Methods section of the manuscript.

3.Role of funder statement: Upon verification, the funder of this study (Ningxia Natural Science Foundation, grant number 2023AAC03543) provided only financial support and had no involvement or influence in the study design, data collection and analysis, decision to publish, or preparation of the manuscript. Therefore, the following statement has been added to the revised cover letter:

“The funders had no role in study design, data collection and analysis, decision to publish, or preparation of the manuscript.”

4.Supporting Information captions: We have now added complete captions for all Supporting Information files at the end of the manuscript (after the References section) and have verified that all in‑text citations to these files match the captions.

Please do not hesitate to contact us if any further changes are required.

Reviewer #1:

Comment 1: Since the work emphasizes binding affinity, a table to show the result of the binding affinities and their corresponding values would improve the result session.

Response:Thank you for this constructive suggestion. We agree that a clear presentation of binding affinity values is very important for the results section. Following the reviewer’s advice, we have now presented the binding affinity values in Fig. 5, which summarizes all binding affinities and their corresponding values. We believe this revision significantly improves the clarity and persuasiveness of our results.

Comment 2: Toxicological relevance and dose considerations

The interpretation of the docking and simulation results should be framed within the fundamental toxicological principle that “the dose makes the poison.” While insilico approaches are valuable for predicting potential molecular interactions, they do not inherently reflect physiological exposure conditions. Importantly, artificial sweeteners are typically consumed at relatively low concentrations and are used in amounts often below those required to drive significant biochemical perturbations in vivo. Additionally, unlike reducing sugars, they are less likely to participate in non-enzymatic glycation reactions and the formation of advanced glycation end products (AGEs).The authors should therefore contextualize their findings within realistic exposure levels, clearly distinguishing between theoretical binding potential and biological risk.

Response:Thank you very much for this important and insightful comment. We fully agree with the reviewer that toxicological conclusions must be grounded in the principle that “the dose makes the poison,” and that our in silico results should be interpreted with careful consideration of realworld exposure levels. In response to this valuable suggestion, we have added a new paragraph in the Discussion section to explicitly address the doserelevance of our findings. In this paragraph, we acknowledge that although our docking and simulation results suggest potential molecular interactions, the actual in vivo significance is contingent upon the achievable local and systemic concentrations of artificial sweeteners under typical consumption patterns. We cite published data indicating that these concentrations are generally low (often in the micromolar or sub micromolar range) and are unlikely to drive the strong biochemical perturbations suggested by the raw binding affinity values.These changes can be found on Page 21, Lines 585–591 (the newly added paragraph in the Discussion section). Once again, we thank the reviewer for guiding us to improve the scientific rigor of our manuscript.

Comment 3: Integration of metabolism and microbiota (key mechanistic gap)

The manuscript does not consider or mention anything about the metabolism of the investigated sweeteners, which is critical for accurate toxicological and physiological interpretation. The biological effects of these compounds are often determined not only by the parent molecules but also by their metabolic fate and biotransformation products.Furthermore, emerging evidence suggests that certain artificial sweeteners may influence host metabolism indirectly through modulation of gut microbiota composition and function. Such alterations can affect key metabolic pathways, including short-chain fatty acid production, glucose homeostasis, and inflammatory signaling.A more comprehensive discussion integrating metabolic pathways of the sweeteners, potential bioactive metabolites, and microbiota-mediated mechanisms would substantially enhance the physiological relevance of the study and provide a more balanced interpretation of the simulation results.

Response:Thank you very much for pointing out this critical mechanistic gap. We fully agree with the reviewer that a comprehensive toxicological and physiological interpretation requires consideration of both the metabolism of the parent sweeteners and the potential modulatory effects of the gut microbiota. In response to this valuable suggestion, we have made the following revisions to the manuscript: We have added a new paragraph in the Discussion section that explicitly addresses the metabolic fate of the investigated sweeteners. We now discuss that many of these compounds are poorly metabolized in humans but may undergo limited biotransformation in the gut or liver. We also discuss that artificial sweeteners can alter the composition and function of the gut microbiota, leading to changes in short chain fatty acid production, glucose homeostasis, and inflammatory signaling. Furthermore, we state that future studies should integrate metabolic profiling and microbiota analysis to translate our computational predictions into a more complete risk assessment framework. These changes can be found on Page 21, Lines 568–585 (the newly added paragraph in the Discussion section). Once again, we thank the reviewer for guiding us to fill this key mechanistic gap.

Reviewer #2:

Comment 1: Transparency in the screening of targets for network pharmacology needs to be improved

The abstract and main text do not specify the source database for sweetener targets, the screening thresholds, or the criteria used to define the 35 “core targets.” The transparency of this process directly affects the reproducibility of the results. It is recommended to provide a detailed list of the databases used and the screening workflow, and to include a brief justification or sensitivity analysis for key thresholds to strengthen the basis of the predictions.

Response:Thank you for raising this important issue regarding transparency and reproducibility. We fully agree that the screening process for network pharmacology targets must be clearly documented. In our original manuscript, we did not provide sufficient detail on the database sources, screening thresholds, or the criteria used to define the 35 core targets. We have now thoroughly revised this section. Specifically, we have described in the Methods section all the databases used to retrieve sweetener related targets, and we have provided a detailed description of the screening process for the core targets (Page 4, Lines 94–105). We thank the reviewer for guiding us to improve the transparency and reproducibility of our analysis.

Comment 2: Presentation of molecular docking results should avoid selective bias

The abstract repeatedly emphasizes the prominent binding potential of luo han guo glycosides and stevioside, yet fails to disclose the total number of tested sweeteners and the distribution of docking scores, which may create the misleading impression that “only these two sweeteners pose a risk.” It is recommended to use a supplementary table to comprehensively list the binding energies of each sweetener with all core targets and to objectively rank risks based on a unified docking score threshold.

Response:Thank you for this valuable and constructive comment. We fully agree that selectively presenting docking results could lead to bias, and we apologize for any misleading impression caused by the original wording. To address this issue and improve the transparency and objectivity of our analysis, we have now listed the binding energies of each sweetener with all core targets in Fig. 5. This table allows readers to view the complete distribution of docking scores and to compare across different sweetener–target pairs. We believe these revisions fully address the concern regarding selective bias and provide a transparent, comprehensive, and objective presentation of the docking results. We thank the reviewer for guiding us to improve the scientific integrity of our manuscript.

Comment 3: Key methodological details of molecular dynamics simulations must be supplemented

The reliability of the conclusions regarding “binding stability” is highly dependent on simulation parameters. Please provide the simulation duration, force field, temperature and pressure conditions, number of independent repetitions, and stability assessment metrics. If necessary, provide RMSD/RMSF plots to fully support the conclusions regarding binding stability with FASN, NOS2, and PCSK9.

Response:Thank you for pointing out the need to provide more detailed methodological information for the molecular dynamics simulations. We fully agree that the reliability of the binding stability conclusions depends on transparent reporting of simulation parameters. In the original manuscript, these details were insufficiently described. We have now provided RMSD/RMSF plots in Fig. 7 to support the binding stability conclusions for FASN, NOS2, and PCSK9. We believe these additions fully address the reviewer’s concerns and provide a solid basis for our conclusions on binding stability.

Comment 4: Incorporate the in vivo metabolism of sweeteners and tissue exposure concentrations into the discussionMolecular docking is based on parent molecules, whereas some sweeteners (e.g., stevioside) undergo significant metabolic transformation in vivo, and actual exposure concentrations in bone and joint tissues are far lower than those in the simulated system. It is recommended to use predictive ADME tools to estimate metabolite and tissue distribution, or to clearly define the physiological concentration assumptions underlying the current “binding potential” in the discussion, thereby making the conclusions more cautious.

Response:Thank you for raising this critical question regarding the in vivo relevance of our docking results. We fully agree that molecular docking based solely on parent molecules, without consideration of metabolic transformation and actual tissue exposure concentrations, may overestimate physiological significance. This is an important limitation that we have now addressed in the revised manuscript. Specifically, we have added a new paragraph in the Discussion section stating that toxicological conclusions must be grounded in the principle that “the dose makes the poison,” and that our in silico results should be interpreted with careful consideration of realistic exposure levels. We also specifically note that actual exposure concentrations in bone and joint tissues are likely far lower than the concentrations used in our simulations, further reducing the physiological relevance of the raw binding scores.These changes can be found on Page 21, Lines 585–591 (the newly added paragraph in the Discussion section). Once again, we thank the reviewer for guiding us to improve the scientific rigor of our manuscript.

Comment 5: Specific evidence is required to establish the association between core targets and musculoskeletal tissues.Inflammatory responses and metabolic disorders are broad pathways and are insufficient to establish specific links to diseases such as intervertebral disc degeneration and myositis. It is recommended to integrate tissue expression profile data from sources such as GTEx to elucidate the expression levels and functional weights of the 35 core targets in tissues such as bone, cartilage, and muscle, thereby strengthening the disease relevance of these targets.

Response:We acknowledge that tissue specific expression data (e.g., from GTEx) would strengthen the association. While we have not performed such analysis in the current study due to resource limitations, we have added a limitation in the Discussion stating that future studies should incorporate tissue expression profiling to validate target relevance in bone/cartilage/muscle.

Comment 6: Network analysis of the four diseases should be conducted using a stratified approach

Although intervertebral disc degeneration, myositis, osteoarthritis, and osteoporosis all fall under the musculoskeletal category, they exhibit distinct molecular pathological differences. Combined analysis may dilute disease-specific signals. It is recommended to supplement the analysis with separate enrichment results for each disease or to employ disease-specific network comparisons to reveal potential differential patterns of sweetener effects across different diseases.

Response:Thank you for this insightful and methodologically important comment. We fully agree that although the four diseases (intervertebral disc degeneration, myositis, osteoarthritis, and osteoporosis) all fall under the musculoskeletal category, they exhibit distinct molecular pathological differences. Using the intersection of sweetener related targets with the

Attachment Submitted filename: Response to Reviewers.docx

# Peer review: Decision Letter 1

**DOI:** 10.1371/journal.pone.0347282.r003

**Authors:** Laxit K Bhatt

2 Jul 2026

Dear Dr. Yuan,

Thank you for submitting your manuscript to PLOS ONE. After careful consideration, we feel that it has merit but does not fully meet PLOS ONE’s publication criteria as it currently stands. Therefore, we invite you to submit a revised version of the manuscript that addresses the points raised during the review process.

Please submit your revised manuscript by Aug 16 2026 11:59PM. If you will need more time than this to complete your revisions, please reply to this message or contact the journal office at plosone@plos.org. When you're ready to submit your revision, log on to https://www.editorialmanager.com/pone/ and select the 'Submissions Needing Revision' folder to locate your manuscript file.

- A letter that responds to each point raised by the academic editor and reviewer(s). You should upload this letter as a separate file labeled 'Response to Reviewers'.
- A marked-up copy of your manuscript that highlights changes made to the original version. You should upload this as a separate file labeled 'Revised Manuscript with Track Changes'.
- An unmarked version of your revised paper without tracked changes. You should upload this as a separate file labeled 'Manuscript'.

If you would like to make changes to your financial disclosure, please include your updated statement in your cover letter. Guidelines for resubmitting your figure files are available below the reviewer comments at the end of this letter.

If applicable, we recommend that you deposit your laboratory protocols in protocols.io to enhance the reproducibility of your results. Protocols.io assigns your protocol its own identifier (DOI) so that it can be cited independently in the future. For instructions see: https://journals.plos.org/plosone/s/submission-guidelines#loc-laboratory-protocols. Additionally, PLOS ONE offers an option for publishing peer-reviewed Lab Protocol articles, which describe protocols hosted on protocols.io. Read more information on sharing protocols at https://plos.org/protocols?utm_medium=editorial-email&utm_source=authorletters&utm_campaign=protocols.

As the corresponding author, your ORCID iD is verified in the submission system and will appear in the published article. PLOS supports the use of ORCID, and we encourage all coauthors to register for an ORCID iD and use it as well. Please encourage your coauthors to verify their ORCID iD within the submission system before final acceptance, as unverified ORCID iDs will not appear in the published article. *Only*  the individual author can complete the verification step; PLOS staff *cannot*  verify ORCID iDs on behalf of authors.

We look forward to receiving your revised manuscript.

Kind regards,

Laxit K Bhatt

Academic Editor

PLOS One

Journal Requirements:

If the reviewer comments include a recommendation to cite specific previously published works, please review and evaluate these publications to determine whether they are relevant and should be cited. There is no requirement to cite these works unless the editor has indicated otherwise.

Please review your reference list to ensure that it is complete and correct. If you have cited papers that have been retracted, please include the rationale for doing so in the manuscript text, or remove these references and replace them with relevant current references. Any changes to the reference list should be mentioned in the rebuttal letter that accompanies your revised manuscript. If you need to cite a retracted article, indicate the article’s retracted status in the References list and also include a citation and full reference for the retraction notice.

[Note: HTML markup is below. Please do not edit.]

Reviewers' comments:

Reviewer's Responses to Questions

**Comments to the Author**

Reviewer #2: All comments have been addressed

Reviewer #4: (No Response)

**********

2. Is the manuscript technically sound, and do the data support the conclusions?

Reviewer #2: Yes

Reviewer #4: Yes

**********

3. Has the statistical analysis been performed appropriately and rigorously?

Reviewer #2: Yes

Reviewer #4: Yes

**********

4. Have the authors made all data underlying the findings in their manuscript fully available?

The PLOS Data policy

Reviewer #2: No

Reviewer #4: Yes

**********

5. Is the manuscript presented in an intelligible fashion and written in standard English?

Reviewer #2: No

Reviewer #4: Yes

**********

Reviewer #2: # Review Comments to the Author

This manuscript delivers solid and meaningful research progress in its corresponding research domain, with a well-defined and innovative research topic that effectively fills existing research gaps in this field. The authors systematically sorted out relevant previous literature, clearly illustrated the research background, research objectives and expected research contributions at the beginning of the article. The whole experimental scheme is reasonable and complete, multiple validated experimental techniques were applied to support the core research conclusion, and all original data and supplementary figures are sufficient and credible. The discussion section thoroughly interprets the experimental findings, objectively analyzes the limitations of this study, and puts forward feasible directions for subsequent follow-up research. The manuscript has clear chapter logic, standardized figure formatting and fluent academic writing, with only trivial grammatical expression and reference format issues to adjust. No serious scientific defects, ethical risks or repeated publication problems are found in this work. Overall, this paper satisfies the publication criteria of this journal, and I strongly recommend accepting this manuscript after minor revisions.

Reviewer #4: Dear authors,

Thanks for the work on addressing the comments from the last round of review, especially on the methodology and discussion sections. Even though the quality of the draft has improved substantially, there are still some minor text suggestions to improve the written quality of the manuscript before publication, most of which are carried from comments not addressed in the previous review comments:

- Minor text edits. There are still several phrases that lack a space after the period in the text (some examples in lines 126, 156, 172,183, 189, and so on...). Please re-review ALL the draft text thoroughly and carefully to ensure this consistent error is corrected in the text, figure captions, and supplemental material.

- Line 56. "Notably, Some existing studies on....". Correct the uppercase "S" in "some".

- Italics are still missing in the CLEAN draft text for the species naming and other term naming (i.e., "homo sapiens", "mus musculus", "in vivo", "in vitro", etc.). Nevertheless, in the markdown version, this error seems to be corrected, but not consistently. Please re-review ALL the draft text thoroughly and carefully to ensure this is corrected in the draft text, figure captions, and supplemental material.

- p-value should be named with lowercase “p”. Nevertheless, there are several “P-value” mentions that need to be corrected to “p-value” throughout the text. Please re-review ALL the draft text thoroughly and carefully to ensure this issue is corrected in the draft text, figure captions AND supplemental material texts.

Your work will increase its quality after these minor edits are implemented. Best of luck with the next stages of publication.

**********

what does this mean?). If published, this will include your full peer review and any attached files.

If you choose “no”, your identity will remain anonymous but your review may still be made public.

**Do you want your identity to be public for this peer review?**  For information about this choice, including consent withdrawal, please see our Privacy Policy

Reviewer #2: No

Reviewer #4: **Yes:**

**********

[NOTE: If reviewer comments were submitted as an attachment file, they will be attached to this email and accessible via the submission site. Please log into your account, locate the manuscript record, and check for the action link "View Attachments". If this link does not appear, there are no attachment files.]

To ensure your figures meet our technical requirements, please review our figure guidelines: https://journals.plos.org/plosone/s/figures

You may also use PLOS’s free figure tool, NAAS, to help you prepare publication quality figures: https://journals.plos.org/plosone/s/figures#loc-tools-for-figure-preparation.

NAAS will assess whether your figures meet our technical requirements by comparing each figure against our figure specifications.

# Author comment: Author response to Decision Letter 2

**DOI:** 10.1371/journal.pone.0347282.r004

6 Jul 2026

Dear Editor and Reviewers,

We sincerely thank the editor and the reviewers for their time and constructive comments on our manuscript. We have carefully addressed each point raised, and the detailed revisions are outlined below. All changes in the revised manuscript have been highlighted using the "Track Changes" mode in Microsoft Word for easy reference. We believe these revisions have substantially improved the quality of our work, and we hope the manuscript now meets the standards for publication in PLOS ONE.

Response to Reviewer #2:

Reviewer’s comment:This manuscript delivers solid and meaningful research progress in its corresponding research domain, with a well-defined and innovative research topic that effectively fills existing research gaps in this field. The authors systematically sorted out relevant previous literature, clearly illustrated the research background, research objectives and expected research contributions at the beginning of the article. The whole experimental scheme is reasonable and complete, multiple validated experimental techniques were applied to support the core research conclusion, and all original data and supplementary figures are sufficient and credible. The discussion section thoroughly interprets the experimental findings, objectively analyzes the limitations of this study, and puts forward feasible directions for subsequent follow-up research. The manuscript has clear chapter logic, standardized figure formatting and fluent academic writing, with only trivial grammatical expression and reference format issues to adjust. No serious scientific defects, ethical risks or repeated publication problems are found in this work. Overall, this paper satisfies the publication criteria of this journal, and I strongly recommend accepting this manuscript after minor revisions.

Response:We sincerely thank the reviewer for the highly positive and encouraging evaluation of our work. The reviewer's recognition that our research topic is innovative, the experimental design is reasonable, the data are sufficient, and the discussion is thorough is greatly appreciated. We also take seriously the reviewer’s suggestions regarding minor grammatical expression and reference formatting issues. In this revision, we have carefully copy-edited the entire manuscript to improve language accuracy and fluency, and we have meticulously checked and corrected all references to conform to the journal’s formatting requirements.We are deeply grateful for the reviewer’s time and valuable guidance.

Response to Reviewer #4�Reviewer’s comment:Thanks for the work on addressing the comments from the last round of review, especially on the methodology and discussion sections. Even though the quality of the draft has improved substantially, there are still some minor text suggestions to improve the written quality of the manuscript before publication, most of which are carried from comments not addressed in the previous review comments:

- Minor text edits. There are still several phrases that lack a space after the period in the text (some examples in lines 126, 156, 172,183, 189, and so on...). Please re-review ALL the draft text thoroughly and carefully to ensure this consistent error is corrected in the text, figure captions, and supplemental material.

- Line 56. "Notably, Some existing studies on....". Correct the uppercase "S" in "some".

- Italics are still missing in the CLEAN draft text for the species naming and other term naming (i.e., "homo sapiens", "mus musculus", "in vivo", "in vitro", etc.). Nevertheless, in the markdown version, this error seems to be corrected, but not consistently. Please re-review ALL the draft text thoroughly and carefully to ensure this is corrected in the draft text, figure captions, and supplemental material.

- p-value should be named with lowercase “p”. Nevertheless, there are several “P-value” mentions that need to be corrected to “p-value” throughout the text. Please re-review ALL the draft text thoroughly and carefully to ensure this issue is corrected in the draft text, figure captions AND supplemental material texts.

Your work will increase its quality after these minor edits are implemented. Best of luck with the next stages of publication.

Response:We sincerely thank the reviewer for taking the time to re-evaluate our manuscript and for carefully pointing out these detailed textual issues. We highly value these suggestions and have addressed each one as follows:

1. Regarding the missing spaces after periods: We have thoroughly and meticulously reviewed the entire manuscript, including all figure captions and supplemental material, and have corrected all instances where a space was missing after a period (including at lines 126, 156, 172, 183, 189, and elsewhere as noted by the reviewer).

2. Regarding the uppercase "S" in "Some" at Line 56: We have corrected this to lowercase "some". The sentence now reads: "Notably, some existing studies on...".

3. Regarding the missing italics for species names and Latin terms: We have systematically reviewed the entire manuscript, all figure captions, and supplemental material to ensure that all species names (e.g., Homo sapiens, Mus musculus) and Latin terms (e.g., in vivo, in vitro) are now consistently italicized throughout.

4. Regarding the capitalization of "p-value": We have uniformly corrected all instances of "P-value" to "p-value" throughout the main text, figure captions, and supplemental material.

We are truly grateful for the reviewer's meticulous reading and valuable suggestions. We believe these revisions have further enhanced the written quality of our manuscript.

Sincerely,

Haifeng Yuan, on behalf of all authors

Attachment Submitted filename: Response_to_Reviewers_auresp_2.docx

# Peer review: Decision Letter 2

**DOI:** 10.1371/journal.pone.0347282.r005

**Authors:** Laxit K Bhatt

17 Jul 2026

A Report on the Mechanisms of Sweeteners in Four Musculoskeletal Disorders: Insights from Network Toxicology, Molecular Docking, and Molecular Dynamics Simulations

PONE-D-26-15470R2

Dear Dr. Yuan,

We’re pleased to inform you that your manuscript has been judged scientifically suitable for publication and will be formally accepted for publication once it meets all outstanding technical requirements.

Within one week, you’ll receive an e-mail detailing the required amendments. When these have been addressed, you’ll receive a formal acceptance letter and your manuscript will be scheduled for publication.

An invoice will be generated when your article is formally accepted. Please note, if your institution has a publishing partnership with PLOS and your article meets the relevant criteria, all or part of your publication costs will be covered. Please make sure your user information is up-to-date by logging into Editorial Manager at Editorial Manager® and clicking the ‘Update My Information' link at the top of the page. For questions related to billing, please contact billing support.

If your institution or institutions have a press office, please notify them about your upcoming paper to help maximize its impact. If they’ll be preparing press materials, please inform our press team as soon as possible -- no later than 48 hours after receiving the formal acceptance. Your manuscript will remain under strict press embargo until 2 pm Eastern Time on the date of publication. For more information, please contact onepress@plos.org.

Kind regards,

Laxit K Bhatt

Academic Editor

PLOS One

Additional Editor Comments (optional):

Reviewers' comments:

Reviewer's Responses to Questions

**Comments to the Author**

Reviewer #4: All comments have been addressed

**********

2. Is the manuscript technically sound, and do the data support the conclusions?

Reviewer #4: Yes

**********

3. Has the statistical analysis been performed appropriately and rigorously?

Reviewer #4: Yes

**********

4. Have the authors made all data underlying the findings in their manuscript fully available?

The PLOS Data policy

Reviewer #4: Yes

**********

5. Is the manuscript presented in an intelligible fashion and written in standard English?

Reviewer #4: Yes

**********

Reviewer #4: Dear authors,

Thanks for the hard work on this paper and for addressing the comments from the second round of review. The quality of the draft has improved since the minor text suggestions have been applied and the draft has been thoroughly re-reviewed.

There are no more comments from my end on the final version of this text. I think, overall, that this paper satisfies the publication criteria of this journal, and I strongly recommend this article for publication.

**********

what does this mean?). If published, this will include your full peer review and any attached files.

If you choose “no”, your identity will remain anonymous but your review may still be made public.

**Do you want your identity to be public for this peer review?**  For information about this choice, including consent withdrawal, please see our Privacy Policy

Reviewer #4: **Yes:**  Dr. Carlos Diaz-Tufinio

**********

# Editor report: Acceptance letter

**DOI:** 10.1371/journal.pone.0347282.r006

**Authors:** Laxit K Bhatt

PONE-D-26-15470R2

PLOS One

Dear Dr. Yuan,

I'm pleased to inform you that your manuscript has been deemed suitable for publication in PLOS One. Congratulations! Your manuscript is now being handed over to our production team.

At this stage, our production department will prepare your paper for publication. This includes ensuring the following:

* All references, tables, and figures are properly cited

* All relevant supporting information is included in the manuscript submission,

* There are no issues that prevent the paper from being properly typeset

You will receive further instructions from the production team, including instructions on how to review your proof when it is ready. Please keep in mind that we are working through a large volume of accepted articles, so please give us a few days to review your paper and let you know the next and final steps.

Lastly, if your institution or institutions have a press office, please let them know about your upcoming paper now to help maximize its impact. If they'll be preparing press materials, please inform our press team within the next 48 hours. Your manuscript will remain under strict press embargo until 2 pm Eastern Time on the date of publication. For more information, please contact onepress@plos.org.

You will receive an invoice from PLOS for your publication fee after your manuscript has reached the completed accept phase. If you receive an email requesting payment before acceptance or for any other service, this may be a phishing scheme. Learn how to identify phishing emails and protect your accounts at https://explore.plos.org/phishing.

If we can help with anything else, please email us at customercare@plos.org.

Thank you for submitting your work to PLOS ONE and supporting open access.

Kind regards,

PLOS ONE Editorial Office Staff

on behalf of

Mr. Laxit K Bhatt

Academic Editor

PLOS One
